# The impacts associated with having ADHD: an umbrella review

**DOI:** 10.3389/fpsyt.2024.1343314

**Published:** 2024-05-21

**Authors:** Blandine French, Gamze Nalbant, Hannah Wright, Kapil Sayal, David Daley, Madeleine J. Groom, Sarah Cassidy, Charlotte L. Hall

**Affiliations:** ^1^NIHR MindTech MedTech Cooperative, Nottingham National Institute of Health Research Biomedical Research Center (NIHR BRC), Institute of Mental Health, School of Medicine, University of Nottingham, Nottingham, United Kingdom; ^2^School of Psychology, University of Nottingham, Nottingham, United Kingdom; ^3^Lifespan and Population Health Unit, University of Nottingham, Nottingham, United Kingdom; ^4^Nottingham Trent University (NTU) Psychology, School of Social Science, Nottingham Trent University, Nottingham, United Kingdom

**Keywords:** ADHD, umbrella review, long-term outcomes, risks, impact

## Abstract

**Introduction:**

Attention Deficit Hyperactivity Disorder (ADHD) affects up to 5% of the population and is characterised by symptoms of impulsivity, hyperactivity and inattention. These symptoms are significantly impairing and carry additional risks for children and adults with ADHD, including negative mental health (e.g. depression), physical health (e.g. obesity) and societal outcomes (e.g. imprisonment, divorce). Very few studies have attempted to synthesise these risks in one publication due to the breadth of evidence published on the adverse outcomes of ADHD.

**Methods:**

An umbrella review was performed to identify reviews (systematic, meta-analysis and narrative) that investigate the risks arising from having ADHD. We conducted a narrative synthesis of the findings and conducted a quality review of the included publications.

**Results:**

Upon searching five databases, 16,675 records were identified. Of these, 125 reviews met the criteria for inclusion. A narrative synthesis of these findings highlighted three key domains of risks associated with ADHD: mental health, physical health, social and lifestyle. Most reviews were of good and moderate quality.

**Discussion:**

This review highlights the many risks associated with having ADHD, beyond its three key symptom domains and the impact of the condition on daily functioning.

**Registration:**

International Prospective Register of Systematic Reviews (PROSPERO CRD42023404073).

## 1 Introduction

Attention deficit hyperactivity disorder (ADHD) is a neurodevelopmental disorder affecting around 5% of children, with symptoms often continuing into adulthood (1). The three key symptoms of ADHD include hyperactivity, impulsivity and inattention, which are developmentally atypical and functionally impairing across at least two settings such as home life and school/work (2). ADHD is associated with differences in cognitive function including impairments in attention, problem-solving, vigilance, inhibitory control, language processing, memory and flexibility (3–6). Other functions such as sensory processing (7), motor skills (8), social skills (9, 10) and emotion regulation (11) can also be affected. These impairments have significant impacts on multiple aspects of life and carry many associated risks. Additionally, ADHD has been linked to comorbid disorders such as sleep disorders (12), learning and mood disorders (13), and other mental health (14) and neurodevelopmental disorders (15). ADHD is recognised as a lifelong condition that has a profound impact on daily functioning and quality of life (16).

The symptoms and impairments associated with ADHD can impact individuals throughout their lifetime and place them at increased risk of poor outcomes. Risks can be defined in many ways but in the context of this review, risks encompass any outcomes that adversely affect the individual and their peers/family, but are not part of the core ADHD symptomatology.

In children and young adults (CYA), many studies have highlighted the health risks associated with ADHD. In a recent cohort study of young people (16–25), Langley and colleagues (17) showed that ADHD was associated with increased risks of anxiety/depression, self-harm, alcohol and drug use and emergency department service use.

ADHD also impacts CYA’s education outcomes. ADHD is associated with increased use of school-based services, increased rates of detention and expulsion, and lower rates of high school graduation and postsecondary education (18). Children with ADHD fare worse than non-ADHD peers on a wide range of additional educational outcomes including: academic attainment, unauthorised absence, exclusion and age of leaving (19). ADHD has also been linked with difficult and antisocial behaviour. There is evidence of relationships between ADHD and delinquent behaviour, adolescent arrest and convictions (20, 21) and these effects are mediated by ADHD symptom severity (22). The prevalence of ADHD in teenage offenders in prison is above the population prevalence, ranging between 4% and 72% (23, 24).

Many health-related risks have also been associated with ADHD in CYA with for example, conditions such as obesity (26), binge eating (27) and Type 1 diabetes (28). ADHD is also associated with higher risks of self-harm (29), early tobacco, alcohol and marijuana use (30, 31) as well as early risky sexual behaviours (32). For example, adolescents with ADHD are at much higher risk of teen pregnancy (33). Although fewer studies have examined later-life impacts, new evidence is showing an increased risk of developing neurodegenerative disease (34). As well as evidence of comorbidities with mental health disorders such as depression and anxiety (35, 36), ADHD has been consistently linked with poorer multiple mental health outcomes (37, 38) in CYA. These risks have significant impacts on day-to-day life but also on CYA’s experiences and quality of life. Compared with non-ADHD children, children with ADHD have reported reduced quality of life, lower happiness, and elevated levels of bullying from siblings (39). Similarly, a recent review (40) demonstrated a consistent positive association between bullying, ADHD in youth and depressive symptoms.

These risks not only affected CYA but also the wider family. Harpin (41) highlights the many impacts that ADHD difficulties have on families and siblings, including the amount of time spent with parents, family activities, more fights with siblings and parents and general family dynamics. Parents report reduced quality and hours of sleep, poorer mental well-being and lower quality of life (42). Additionally, parents of CYA with ADHD report higher levels of stress due to the difficulties experienced by their children and the impact these have on the family (43, 44). Peasgood and colleagues (39) found that siblings of children with ADHD also reported lower quality of life.

While many risks are associated with ADHD in childhood, adult ADHD has also been associated with many adverse health outcomes, including smoking and substance abuse, poor sleep, physical injury, obesity, hypertension and diabetes. Nigg (45) also reported that ADHD was associated with an increased risk of cardiovascular disease including cardiac arrest, stroke and vascular disease.

Many links with psychological health have also been made. ADHD has been linked with worse mental health outcomes such as depression (46) or anxiety (47) and often lower self-esteem (48) and quality of life (49). A recent review found that the most frequent psychiatric disorders comorbid with ADHD were substance use disorders, mood disorders, anxiety disorders and personality disorders (50). Unfortunately, these mental health risks can lead to more extreme outcomes such as suicide. Garaz and Balazs’s (51) systematic review of longitudinal studies showed a positive association between the presence of ADHD diagnosis in childhood and suicidal thoughts and/or attempts in adulthood. Septier and colleagues (52) also demonstrated a significant association between ADHD and suicidal attempts, suicidal ideations, suicide plans and completed suicide. Additionally, ADHD has been significantly associated with early mortality and reduced life expectancy (53).

As well as health risks, many other functional impairments have been established. These include driving risks, accidents, impairments (54, 55), lower academic achievements, lower full-time employment and household income (56) increased social impairments (57) or higher risks of divorce (58). ADHD is also associated with higher rates of gambling (59), and a five-fold increased rate of imprisonment compared to the general population (60). Reinhardt and Reinhardt (61) reviewed the risks associated with ADHD and observed several situations in which ADHD was the most relevant psychiatric diagnosis in relation to urgency (a specific aspect of impulsivity) including higher rates of accidents, suicide, exposure to violence or sexual abuse.

The impacts of having ADHD have been widely researched and documented but to date, only a few reviews have attempted to summarise these in a comprehensive synthesis, all with their own limitations (62–65).

Shaw and colleagues (65) reviewed the long-term outcomes of having ADHD and found that these outcomes affected key domains related to: addictive behaviour, academic difficulties, antisocial behaviour, social function, occupation, self-esteem, driving and obesity. They demonstrated that adults with ADHD experienced poorer outcomes in all these domains with the most often studied outcomes being substance abuse, academic difficulties and antisocial behaviour. However, this review focussed primarily on the effect of treatment on these outcomes.

Ginsberg and colleagues (64) evaluated the impacts of underdiagnosis and undertreatment of ADHD through their review. They demonstrated that ADHD had effects on multiple outcomes including educational and vocational underachievement, social interactions, antisocial behaviour, substance abuse, imprisonment, driving, and physical and psychiatric comorbidities (such as eating disorders, anxiety, phobia, depression, sleep disorders). While this review usefully measured a wide range of outcomes, its main focus was on underdiagnosis and undertreatment and the search strategy was limited to one database, limiting the reliability of the findings.

Di Lorenzo and colleagues (63) looked at prospective studies of the long-term outcomes of children and adolescents with ADHD. They found that ADHD was associated with five key constructs, namely: substance abuse, antisocial behaviour, criminal activities, anxiety and depression. However, this review limited the scope of the findings by only including studies with a 5-year (or less) outcome timeframe, meaning that longer-term risks may have been missed.

Cherkasova and colleagues (62) also looked at prospective studies from childhood to adulthood on the long-term outcomes of ADHD in adulthood. These outcomes included: impairments in education and occupation (lower educational attainment and lower occupational status), mental health comorbidities and suicide attempts, and physical health (increased mortality, smoking, obesity, poor sleep), substance abuse, driving and antisocial behaviour. However, the inclusion criteria for this review limited the outcomes to only seven included studies.

The breadth of publication on this topic is significant and it would be extremely difficult to gather all publications on the topic, which is why the reviews above impose strict limitations on their search criteria. However, this might have led to missing key constructs around this important topic. The present review proposes a novel approach to synthesise the impact of having ADHD by conducting a review of reviews. Only reviews of outcomes associated with ADHD were included. This allowed the synthesise of outcomes to be manageable but also to include outcomes that have been evidenced over time through multiple publications, strengthening the validity of these impacts. Additionally, while all previous reviews have focussed on adult outcomes, this review will establish the impacts for both children and adults.

This review aims to synthesise the adverse impacts that ADHD has on children and adults, with regard to physical and mental health, and social and lifestyle functioning.

## 2 Methods

This review was written in accordance with the Preferred Reporting Items for Systematic Reviews and Meta-analysis Protocols (PRISMA-P) guidelines (66). A protocol for this review is registered with the International Prospective Register of Systematic Reviews (PROSPERO; CRD42023404073).

### 2.1 Inclusion criteria

#### 2.1.1 Type of studies

Eligibility criteria included: published reviews of any design (including, but not limited to narrative reviews, systematic reviews (with and without meta-analysis) or scoping reviews). exploring the impacts, long-term outcomes or risks associated with ADHD. Only studies published in peer-reviewed publications were considered.

#### 2.1.2 Type of population

Eligible reviews included individuals (adults and children) who meet the criteria for or have received a diagnosis of ADHD, as defined by the review authors, this criterion was based on a variety of methods including meeting DSM or ICD criteria, self-report, or achieving a specified cut-off on a validated measure. If reviews included multiple groups such as ADHD and autistic individuals, ADHD findings were extracted and reported separately if possible.

#### 2.1.3 Type of phenomenon of interest

This review examined the impacts of having ADHD. Within the context of this study, impacts were defined as any direct consequence of the condition on daily life, encompassing consequences for the individuals, their environment (such as job, schools, friendships), their families and any others impacted. The precise ways in which impact is defined and measured differ between studies. We therefore aimed to capture broader concepts such as risks, effects, outcomes or consequences that transcend different impacts of having ADHD.

#### 2.1.4 Context

Included papers were conducted in any setting and took an international perspective. The period of the review was not restricted, covering all publications from inception up to July 2023.

### 2.2 Exclusion criteria

Unpublished and grey literature was excluded, as were publications that were not peer-reviewed. Reviews were also excluded if they did not specify the status of the neurodevelopmental disorder examined and did not mention the term “ADHD”. Reviews linking impacts to other neurodevelopmental disorders with common (such as autism, dyspraxia etc.) were excluded. Studies that are not reviews were excluded as well as studies not published in English. Reviews on the prevalence, assessment, interventions, management, treatment and treatment outcomes of ADHD were excluded. Reviews on biological features such as brain correlates, genetics, biological mechanisms, cognitive tests, and executive and motor functions were also excluded. Reviews that did not report a direct link between diagnostic status and risks were excluded. Finally, reviews that reported more than one direct link were excluded as it was not possible to separate the direct impacts and reviews on risk factors for ADHD.

### 2.3 Search strategy

Five databases (PsycINFO, Embase, Scopus, Medline, ERIC) were searched. Following this search and removal of duplicates, a preliminary analysis was conducted of the subject headings (MeSH) and text words (in the title, abstract, and author keywords) related to ADHD and risks. PROSPERO was checked for ongoing or already published systematic reviews on the subject. A full search strategy for Medline (MEDLINE In-Process & Non-Indexed Citations and OVID MEDLINE 1946 to present-Ovid) is detailed in **Supplementary Material** as an example. The MEDLINE search strategies were adapted for the other databases according to their individual structures. The search was performed in July 2023, date limits were not imposed. While hand-searching was not a strong component of our planned search strategy, the reference lists of all papers that meet the inclusion criteria were hand-searched to check for any additional reviews.

### 2.4 Study selection

**Figure 1:**
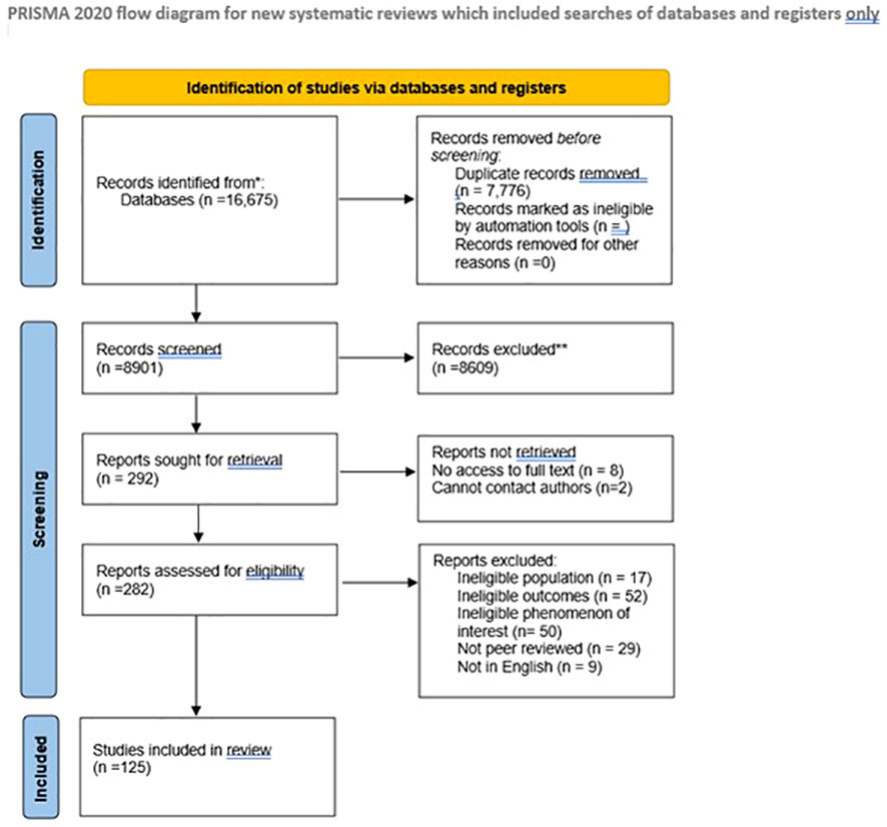
Study selection flow.

Following the search, all identified citations were uploaded into reference manager software (Zotero). Two of the review authors (BF and GM) independently screened the titles and abstracts for assessment against the search inclusion criteria. Full reports were obtained for all titles that appear to meet the inclusion criteria. The same two review authors screened and assessed the full-text reports in detail against the inclusion criteria. Studies that did not meet the inclusion criteria were excluded and a record of reasons for excluding trials is provided. The study selection process is presented below (**Figure 1**).

### 2.5 Data extraction and outcomes

#### 2.5.1 Data extraction

Two reviewers independently extracted qualitative and quantitative data from the included studies.

#### 2.5.2 Outcomes

The main outcome is the synthesis of impacts and risks associated with having ADHD. Multiple types of factors reported in the selected reviews were evaluated such as societal factors (divorce, imprisonment etc.) and health factors (suicide, drug abuse, etc.). These factors were grouped into themes within the synthesis phase.

### 2.6 Data synthesis

Qualitative and quantitative findings were aggregated into a narrative synthesis. The aggregation/configuration of all themes generates a set of statements that represent the final aggregation (or the development of a theoretical framework, a set of recommendations, or conclusions). Two reviewers (CLH and BF) conducted the syntheses in sequential order; one reviewer developed the synthesis and the second checked the findings. Any disagreement was discussed and/or mediated by a third reviewer.

### 2.7 Assessment of methodological quality

Following mixed methods review guidelines (67), two review authors (HW and BF) independently critically appraised all selected reviews for methodological quality. 104 of the reviews were systematic reviews (with or without meta-analysis) and were assessed using a standardised quality appraisal tool by the Joanna Briggs Institute Tool for Systematic Reviews (68). The remaining 21 literature or narrative reviews were scored on the Scale for the Assessment of Narrative Review Articles (SANRA, 69) as it was not appropriate to score these on the systematic review checklist. Any disagreement between reviewers was resolved through discussion and/or a third reviewer. The quality rating of each study did not affect the inclusion of the review; all reviews that met the inclusion criteria were submitted to the data extraction and synthesis process.

## 3 Results

### 3.1 Study selection

The study selection process is shown in the flow chart above (**Figure 1**). Reasons for excluding reviews after full-text assessment are provided in **Supplementary Data Sheet 1**. In total, 125 reviews published between 1991 and 2023 met the inclusion criteria. The reviews included systematic reviews, meta-analyses, narrative reviews, rapid reviews, and scoping reviews. Characteristics of each review and their review themes are given in **Supplementary Table 1** (**Supplementary Material 1**). A range of countries were represented within the reviews, encompassing a worldwide representation.

### 3.2 Data methodological quality

Results of study quality are reported in **Supplementary Table 1**. Following Kmet, Lee and Cook’s guidelines (70), an original quality score from 0 to 1 was calculated for each review. Scores were then classified into poor (0–0.44), moderate (0.45–0.69) and good (0.70–1.00). The same boundaries were used for the scores from the SANRA scale for the non-systematic reviews for consistency in the table. The studies reviewed using SANRA are marked in **Supplementary Table 1**. Study quality was assessed and agreement between reviewers was 88% overall. The studies showed some variation in their quality. Of the 125 studies, 53 (42%) scored ‘good’, 60 (48%) scored ‘moderate’ and 12 (10%) scored ‘poor’.

### 3.3 Data extraction and summary of results

#### 3.3.1 Mental health (42 reviews)

Forty-two reviews highlighted the important association between mental health and ADHD (see **Table 1**). These risks covered a range of topics including addiction, suicide and self-harm, mood, personality, and other disorders.

**Table 1:** Summary of mental health reviews.

Mental health			
Mental health area	Number of reviews	Association	No association
Addiction	16 reviews*: 2 internet addiction 1 gambling 8 substance/alcohol 4 gaming 1 mixed	2 reviews showed an association with internet addiction. 1 shows a positive association with gambling 8 reviews showed a positive association between ADHD and various forms of substance/alcohol addiction (including substance misuse, alcohol and cigarettes) 4 reviews showed a positive association between ADHD and gaming issues. 1 mixed showed a positive association with behavioural addiction (including gaming, sex and internet addiction)	1 of the 8 reviews on substance/alcohol found no association between ADHD and alcohol misuse
Suicide and self-harm	8 reviews	8 reviews showed a positive association between ADHD and suicide or self-harming behaviours. 4 reviews specifically highlight the mediating link of co-morbidities in this relationship	
Mood and personality disorders	8 reviews 1 OCD 7 bipolar and mood disorders	8 reviews showed a positive association with mood disorders but with the caution that the relationship was less evident in longitudinal research. Two reviews showed a positive association in adults with anxiety. The review on OCD showed a link in children but not adults	
Other disorders	7 reviews 4 eating disorders 3 psychotic disorders	6 reviews explored other topics including eating disorders and psychotic disorders. Overall, the reviews indicated a positive relationship between ADHD and these disorders including schizophrenia and anxiety	
Self-esteem	1 review	The review showed a positive association among adults	

*Biederman’s paper covered multiple topics, hence the number of reviews in each topic is more than the sum of the total papers.

##### 3.3.1.1 Addiction

A total of 16 reviews explored various forms of addiction.

Two meta-analyses looked at internet addiction and ADHD in children, young people and adults (71, 72). The findings demonstrated that ADHD was associated with internet addiction (71) and had a positive correlation with more severe symptoms of ADHD (72). One meta-analysis looked at gambling and individuals with problem gambling were 4.18 times more likely to have ADHD than controls. Individuals with ADHD were 2.85 times more likely to experience problem gambling (gambling that disrupts, damages or interferes with daily life) than individuals without ADHD (59).

Eight reviews looked at substance or alcohol disorders/misuse (73–80). All reviews showed a positive association between ADHD and substance misuse (drugs and alcohol). Overall, the findings indicated a prevalence of approximately one-quarter of people with ADHD having substance misuse and similar rates of ADHD in patients with substance misuse (77–79). A review conducted by (76) found ADHD was an independent risk factor for developing substance disorder during childhood and adolescence and found that children with ADHD had twice the risk of developing nicotine, alcohol or substance misuse compared to children without ADHD. Willens (80) also found that ADHD increased the risk of cigarette smoking and that ADHD was associated with greater substance misuse severity and chronicity.

A meta-analysis of 13 studies conducted by Charach (73) showed that children with ADHD are at risk of developing substance and alcohol use disorders. However, the odds ratio (1.35) was lower for alcohol disorder than for nicotine (OR=2.36) or drug use (OR=3.48). The authors state this may be due to the influence of one or two studies within a relatively small pool of papers. Interestingly, Lee (74) found childhood ADHD predicted substance misuse, but not alcohol. Luderer (75) found ADHD was highly prevalent in patients with alcohol use disorder, with 43% of individuals with ADHD developing alcohol-related disorders and approximately 20% of individuals with alcohol disorder having ADHD. Of note, Van Emmerik (79) found that cocaine dependence was associated with lower ADHD prevalence than alcohol dependence. However, their review also highlighted the importance of the choice of measurement scale.

Four reviews looked at gaming (81–84). Three systematic reviews reflected young people (81, 82, 84) and one (83) did not report age, all found an association between ADHD and gambling. Weinstein (83) found a relationship between computer game addiction and ADHD, which they proposed shared a common mechanism of reward and sensitization mediated by dopamine. Gonzalez-Bueso (82) found a high correlation between gaming disorders and ADHD. Dullur (81) found the strongest correlations were with the ADHD inattentive subscale, however, they also noted poor quality studies, predominantly using survey waves or group-wise comparisons. Salerno’s (84) review showed that ADHD symptoms may be a risk factor for problematic gambling, particularly for males. However, although some studies supported a bidirectional correlational relationship between gaming and ADHD symptoms, one of the studies included in the review showed (83) that whereas ADHD predicted more time gaming, gaming behaviour did not predict greater ADHD symptoms.

Finally, one review explored an association between ADHD and various forms of addiction (85) and found an association between ADHD and gambling, sex and internet addiction. The overall prevalence of comorbid ADHD in individuals with addictions ranged from 5.8-88.3% and the prevalence of addiction in individuals with ADHD ranged from 5.9-71.8%.

##### 3.3.1.2 Suicide and self-harm

Eight papers looked at ADHD suicide and self-harm (29, 51, 52, 86–90). All reviews showed a positive association. A meta-analysis conducted by Septier (52) demonstrated a significant association between ADHD and various markers of suicidal behaviour including, suicidal attempts (OR; 2.37, 95% CI = 1.64–3.43; I^2^ = 98.21), suicidal ideations (OR; 3.53, 2.94–4.25; I^2^ = 73.73), and completed suicide (OR; 6.69, 3.24–17.39; I^2^ = 87.53). A review conducted by James (86) showed the overall suicide rate in ADHD was 0.63-0.78%. The authors suggest that the increase may be particularly prominent in males and as a result of worsening of co-morbid conditions. Giupponi (87) also highlighted that it is unclear if the increase in suicidal behaviour reflects a direct link or is due to the worsening of co-morbid conditions. Balazs and Kereszteny (88) found that co-morbid disorders mediated the role between ADHD and suicide while Impey and Heun (89) found that co-morbidities of delinquency and substance misuse had a large influence on the association.

##### 3.3.1.3 Mood and personality disorders

Seven reviews looked at mood disorders (91–97), and an additional review conducted by Biederman (98) also covered mood disorders amongst other topics. Overall, the studies found a positive association between the two disorders, however, there is a need to conduct further longitudinal research.

Schiweck (94) showed an association between ADHD and mood disorders, demonstrating that up to 1/13 individuals with ADHD have bipolar disorder and up to 1 in 6 bipolar patients have ADHD. Sandstrom (97) found ADHD was three times more common in people with mood disorders compared to those without and 1.7 times more common in bipolar compared to mood disorders. Brancati (91) found a greater risk of bi-polar occurrence in ADHD compared to healthy controls (risk ratio: 8.97, 95%-CI: 4.26-18.87) while Zdanowicz and Myslinski (96) cautioned that despite evidence of potential dual diagnosis between ADHD and bi-polar, there is a need to consider them as two separate diagnoses.

Faraone (92) conducted a meta-analysis of family genetic studies and found a significantly higher prevalence of ADHD amongst relatives of bipolar probands (relative risk, RR; RR=2.6; 95% CI=2.1–3.2) and the same pattern emerged for bipolar amongst relatives of ADHD probands, indicating a potential genetic relationship between the two.

Meinzer (93) showed that 22 out of 29 papers revealed a positive relationship between ADHD and depression, however, there was large variability in the association. The link was most evident in cross-sectional studies, but for longitudinal studies, there was weaker/no reliable evidence. Skirrow (95) also indicated a relationship between the two, including a familial link found in genetic studies, however, they also highlighted the lack of consistent evidence from longitudinal studies and the need to conduct further research.

Additionally, one review (99) explored the association between ADHD and OCD. Overall, the paper found an increased risk of OCD with ADHD in children, but with large variability (0-60%). This relationship was not observed in adults, although the authors noted a lack of research in adult populations hampering the ability to draw definitive conclusions. Another looked specifically at the link between ADHD in childhood and later adult Borderline Personality Disorder (100). The review found an association between the two and suggested the need for further research to explore whether ADHD is a specific risk factor for some sub-groups of BPD (e.g. predominately impulsive or predominately affective). Furthermore, the Biederman review (98) also explored the link between BPD and ADHD as well as anxiety and BPD and found a positive association.

##### 3.3.1.4 Other disorders

Four reviews (including one meta-analysis) showed a positive association between ADHD and eating disorders in children and young people (27, 101–103). Nazar (102) found a three-fold increase in ADHD in young people with eating disorders and a two-fold increase in eating disorders in young people with ADHD. However, Nickel (103) found a large variation in the relationship, ranging from no relationship to 21.8% of females with ADHD having ED.

Three reviews (50, 104, 105) (including one meta-analysis) explored the link between ADHD and psychotic disorders, demonstrating a positive association between Schizophrenia and ADHD. Norredine (105) found an increased risk of personality disorders for participants with childhood ADHD with a pooled relative effect of 4.74 (95% CI 4.11-5.46). Choi (50) found in schizophrenia patients, the prevalence of ADHD in childhood ranged from 17-57% and 10-47% in adults.

##### 3.3.1.5 Self-esteem

Although not a mental health disorder, our search revealed one review that looked at ADHD and self-esteem (106). The review was based on the findings from only 13 articles, some of which were of low methodological quality, due to weak design or sample size concerns. However, overall, the review concluded that ADHD was associated with lower self-esteem in adults, which could be partially mitigated by psychotherapeutic interventions.

#### 3.3.2 Physical health (51 reviews)

Fifty-one reviews investigated the risks associated with ADHD and physical health (see **Table 2**). These risks encompass, sleep, oral health, weight, accidents and injuries, and other diseases and impairments. A review of 126 studies investigating the relationship between ADHD and somatic diseases demonstrated that Obesity, sleep disorders, and asthma were well-documented comorbidities with adult ADHD (107). Tentative evidence was found for an association between adult ADHD and migraine and celiac disease and in a large health registry study, cardiovascular disease was not associated with adult ADHD (107).

**Table 2:** Summary of physical health related reviews.

Physical health			
Health area	Number of reviews	Association	No association
Sleep	13 reviews	All reviews showed a significant relationship between sleep difficulties and ADHD.	However, 5 reviews reported caution in generalising findings due to lack of studies, methodological issues and continuity
Oral Health	6 reviews	Six reviews showed a link between poor oral health and habits and children with ADHD.	
Weight	9 reviews	All reviews have shown an association between obesity/overweight and ADHD	The relationship between ADHD and obesity is well established, however, in children, the findings are mixed with 3 studies relaying contractive/weaker results.
Accidents and injuries	8 reviews	Eight reviews showed an increased risk of accidents, injuries and mortality across the life span with significant differences between children and adults	
Other Diseases and impairments	15 reviews	One review showed an association with celiac disease in children Two reviews showed a link with asthma One with some vision impairments Two with restless leg syndrome One on chronic pain One on vision One on type 2 diabetes One on the allergic diseases One on neurodegenerative diseases One on cardiovascular diseases	One review showed no association with celiac disease

##### 3.3.2.1 Sleep

The relationship between sleep and ADHD was explored in 13 different reviews, reporting the findings of over 323 studies (25, 84, 108–119).

Many reviews investigated the link between ADHD and sleep in children. Cortese and colleagues (109), demonstrated in an early review that the number of movements in sleep, and the apnea index were significantly higher in children with ADHD than in controls but no significant differences in sleep-onset difficulties and bedtime resistance between children with ADHD and controls were found after controlling for comorbidity and medication status. In a subsequent review, the same author (110) found that children with ADHD are significantly more impaired in most of the parentally reported sleep items (concerning problematic behaviours around bedtime and in the early morning) as well as in some measures indicating fragmented sleep, poor sleep efficiency, and excessive daytime sleepiness. Additionally, children with ADHD are more likely to experience sleep-disordered breathing (25) and periodic limb movement in sleep (118). Sleep disturbance was also significantly higher in children aged 7-12 with ADHD (117) and in adolescents (116). While both studies highlighted the limited number of methodologically sound studies on this topic, Bondopadhyay and colleagues (108) reviewed over 148 studies and demonstrated that sleep disturbances in ADHD are common and that they may worsen behavioural outcomes.

Reviews focusing on adults only have also demonstrated similar findings. Adults with ADHD are more likely to experience sleep onset latency and poorer sleep efficiency (111, 115), greater number of awakenings during sleep, and a general lower self-perceived sleep quality compared with healthy controls (115). Sleep disorders in adults with ADHD have also been shown to have a bidirectional relationship, with poor sleep exacerbating ADHD symptoms and vice-versa (84).

Finally, a few studies have looked at sleep and ADHD over the lifespan. Kim and colleagues (113) revealed that the prevalence of ADHD symptoms in narcolepsy was 33% (95% CI, 28.0–38.3). This prevalence was higher in adults (36.2%) compared to children and adolescents (25.0%). Short sleep duration has also been shown to be associated with ADHD symptomology, especially hyperactivity, with a subgroup meta-analysis demonstrating a significant correlation in studies where sleeping time was six hours or less (114). Additionally, ADHD has been reported for up to 95% of individuals suffering from obstructive sleep apnea (OSA), demonstrating another bidirectional relationship with OSA potentially contributing to ADHD symptomatology in a subset of patients diagnosed with ADHD (119).

##### 3.3.2.2 Oral health

Six reviews explored the relationship between ADHD and oral health in children (120–125). A meta-analysis of 26 reviews (120) found a significantly higher number of decayed surfaces, higher plaque scores and higher dental trauma risks. Drumond and colleagues (121, 122) also demonstrated a higher chance of dental trauma and that children and adolescents with ADHD had higher gingival inflammations. Despite weaker evidence, Manoharan (124) demonstrated that dental caries were also more common in children with ADHD. Finally, children with ADHD are also more likely to have tooth grinding and clenching (123). This finding was confirmed by Souto-Souza (125) in a meta-analysis of 27 studies that demonstrated a much higher level of sleep and awake tooth grinding and/or clenching in children with ADHD.

##### 3.3.2.3 Weight

Nine reviews explored the relationship between weight and ADHD (126–134), primarily focussing on overweight and obesity and representing over 260 studies.

Multiple reviews by Cortese and colleagues (126–129) demonstrated that obese patients referred to obesity clinics may present with a higher than expected prevalence of ADHD, that individuals with ADHD are heavier than expected (126, 127) and have a higher than average body mass index (127). The pooled prevalence of obesity was increased by about 70% in adults with ADHD (28.2%, 95% CI=22.8–34.4) compared with those without ADHD (16.4%, 95% CI=13.4–19.9), and by about 40% in children with ADHD (10.3%, 95% CI=7.9–13.3) compared with those without ADHD (7.4%, 95% CI=5.4–10.1) (128). Although findings are mixed across individual studies, meta-analytic evidence shows a significant association between ADHD and obesity, regardless of possible confounding factors such as psychiatric comorbidities (129). These findings confirmed that individuals with ADHD are heavier than expected and that the prevalence of ADHD in obese patients may be higher than expected (130), especially in adults (131).

Li and colleagues (132) demonstrated a small overall association between ADHD and obesity in children, but this effect is moderate in adults. This difference between children and adults was also highlighted by another review which found no reliable association between ADHD and body mass index in children at any age or time point (133). In this national survey, ADHD was associated with obesity only in adolescent girls and adults but not in children or boys. However, the most recent review, highlighted different findings showing that children with ADHD had a significant risk for co-occurring overweight and obesity [OR 1.56; 95% confidence intervals (CI) 1.32–1.85], especially in particular groups such as boys (OR 1.45; 95% CI 1.10–1.90), people in Asia (OR 3.25; 95% CI 1.70–6.21) and Europe (OR 1.85; 95% CI 1.61–2.12), and patients not using medication (OR 1.54; 95% CI 1.22–1.94) (134).

##### 3.3.2.4 Accidents and injuries

The link between accidents, injuries and ADHD was observed in eight reviews (135–142).

Reviews focussing on children established many different risks. A significantly higher risk of poisoning has been shown in children and adolescents with ADHD compared with their non-ADHD peers, with an estimated relative risk of 3.14 (95% CI=2.23 to 4.42) (135). Children and adolescents with ADHD are also at an increased risk of unintentional injuries (141) and two times more likely to have bone fractures (142). With regards to brain injuries, severe traumatic brain injuries in children appear to be associated with an increased risk for attention-deficit/hyperactivity disorder compared with non-injured and other injured controls, however no association between ADHD and concussions and mild or moderate traumatic brain injury was identified (138).

While many risks are observed in childhood, many also occur across the lifespan. As opposed to Asarnow’s findings (33), strong evidence for an association between ADHD and mild traumatic brain injury (mTBI) has been demonstrated across the lifespan (136). However, most studies fail to report which came first and therefore the sequencing of ADHD and mTBI must be made with caution (136). A meta-analysis of 35 studies demonstrated that individuals with ADHD were two times more likely to be injured than control (137). The risk of accidents and injuries differs across age groups, peaking in adolescents and young adults with a cluster around ages 12–25 years (139).

Finally, ADHD has been linked to mortality. While we know that poisoning is an important cause of mortality amongst all children, those with ADHD have a higher risk of poisoning and are therefore more at risk of dying through poisoning (135). All-cause mortality was also found to be higher in individuals with ADHD than for the general population, most specifically through deaths from unnatural causes were higher than expected (10 studies; RR, 2.81; 95% CI, 1.73-4.55; I2, 92%; low confidence) (140).

##### 3.3.2.5 Diseases and Impairments

Finally, fifteen reviews reported links between ADHD and multiple diseases and impairments (34, 107, 143–157).

Children-focussed reviews highlighted many significant risks in early years. Children with ADHD are more at risk of having lower urinary tract symptoms (LUTS- urinary frequency, pressure, urgency, and overactive bladder syndrome) with the severity of ADHD positively associated with the severity of LUTS (151). A systematic review investigating allergic diseases showed that children with ADHD had elevated rates of asthma compared to children without ADHD but no association with food allergy and weak links with allergic rhinitis, atopic dermatitis, and allergic conjunctivitis (153). Additionally, an association between chronic pain and ADHD in children has been shown (144). While a review of celiac disease in children showed that children with ADHD were more likely to have celiac disease (149), another review on celiac disease in the lifespan showed no conclusive link with ADHD (147).

Across the lifespan, many risks are associated with diseases and impairments. A review looking at ADHD and vision (143) found evidence of an association between ADHD and reduced colour discrimination, astigmatism, hyperopia and hypermetropia, and strabismus (but not myopia), however, did not detect a higher prevalence of ADHD in patients diagnosed with problems of vision. Although still limited, evidence from clinical and population studies demonstrates an association between restless leg syndrome (RLS) and ADHD or ADHD symptoms across the lifespan (146, 152). In the ADHD group, RLS symptoms ranged from 11 to 42.9% in children and 20–33.0% in adults (152). Individuals with ADHD also experience a two-fold increased risk of developing type two diabetes compared to those without ADHD (Cohen’s d = 0.46, CLES = 62.68%) (148). A positive association between ADHD and migraine has also been reported (155) as well as with celiac disease (147). In ageing populations, a history of ADHD may increase the risk for the development of neurodegenerative diseases, in particular Lewy body diseases (LBD), by up to five-fold (34). Finally, an increased risk of cardiovascular diseases was associated with ADHD across all ages (157).

#### 3.3.3 Social and lifestyle (32 reviews)

Daily life, social life and lifestyle are also strongly affected by having ADHD and was explored in 31 reviews (see **Table 3**). These impacts included risks linked with offending and criminality, education and employment, quality of life, relationships and social interactions and risk-taking behaviour.

**Table 3:** Summary of social and lifestyle reviews.

Social and lifestyle			
Topic area	Number of reviews	Association	No association
Offending and criminality	9 reviews	All showed a positive association between ADHD and offending and criminal behaviour. One also showed a positive association between ADHD and being a victim of crime.	
Employment and education	5 reviews	All reviews showed poor educational and employment outcomes such as fewer attainment, prematurely leaving schools, more frequent changes	
Quality of life	5 reviews	All reviews showed poorer QoL in adults and children	
Relationship and social interactions	5 reviews	All reviews showed poorer social relationships including, peer functioning social skills, social dysfunction, peer interaction and intimate relationships.	
Risk-taking	6* reviews	All 6 reviews showed a positive relationship between ADHD and risk-taking – including pregnancy, driving offences and general risk-taking	2 of the 6 reviews noted no increased risk for vehicle crashes despite the overall risk of increased driving offences
Others	2 reviews	Association between gender dysphoria and ADHD, but limited data of poor quality One review showed an association with problematic internet use	

Six reviews were included in this topic, however, two reviews were the same paper published twice under different titles (158, 159).

##### 3.3.3.1 Offending, criminality and violence

Nine reviews looked at the link between ADHD and criminality (21, 60, 160–166). The findings showed a positive association between ADHD and offending (96, 99).

Having ADHD was associated with an earlier onset and an increased risk of re-offending (21, 165). Mohr (163) presented risk ratios of childhood ADHD and adolescent and adulthood arrests (RR: 2.2, 95% CI: 1.3–3.5), convictions (RR: 3.3, 95% CI: 2.1–5.2) and incarcerations (RR: 2.9, 95% CI: 1.9–4.3). One review found 45% of youths incarcerated screened positive for ADHD (165). A further review (166) using diagnostic interview data, indicated the estimated prevalence of ADHD in prison was 25.5% and there were no significant differences for gender and age. Similar findings were reported by Baggio (60) who found that the adult ADHD prevalence rate for incarnated individuals was 26.2% (95% confidence interval: 22.7–29.6), with retrospective assessments of ADHD in childhood being associated with an increased prevalence estimate (41.1, 95% confidence interval: 34.9–47.2).

Buitelaar (161) specifically explored intimate partner or domestic violence and found positive associations between childhood and/or adult ADHD and adult domestic violence/Intimate Partner Violence. However, they note some studies did not control for comorbid Conduct Disorder (CD) or Antisocial Personality Disorder (ASPD) which may have impacted the findings. Similarly, Arrondo’s (160) meta-analysis showed a higher risk of ADHD individuals being involved in interpersonal violence as perpetrators (six studies, OR 2.5, 95% CI 1.51–4.15) or victims (OR 1.78, 95% CI 1.06–3.0). Additionally, individuals with ADHD were at increased risk of being perpetrators (three studies, OR 2.73, 95% CI 1.35–5.51) or victims of sexual violence (OR 1.84, 95% CI 1.51–2.24).

##### 3.3.3.2 Employment and education

Five reviews established the links between ADHD, employment and education (167–171). Poor education outcomes have been consistently reported in the literature. Academic risks have been demonstrated in various ways such as fewer attainment of a Bachelor’s degree compared to controls (170), poor education performance and prematurely leaving school both at the high school and college level (167), grade repetition, need for special education, lower scores on achievement tests (168), higher risks of school drop-out (171). An extensive review of 176 studies (169) found that achievement test outcomes (79%) and academic performance outcomes (75%) were worse in individuals with untreated ADHD compared with non-ADHD controls, also when IQ difference was controlled. Improvement in both outcome groups was associated with treatment, more often for achievement test scores (79%) than academic performance (42%), also when IQ was controlled (100% and 57%, respectively).

ADHD diagnosis affected the nature of the individual’s employment. Individuals with ADHD are more likely to struggle with work performance and demonstrate difficulty maintaining job stability and attaining high-status jobs, subsequently face more financial hardships and have a greater reliance on public aid than those without ADHD (167). Adults with ADHD are less likely to be in employment, especially full-time, and change work more frequently than controls (171). Christiansen and colleagues (170) highlighted that adults with childhood-diagnosed ADHD, generally experience employment of lower quality compared with peers, in relation to income, education and occupational attainment. Additionally, adults with persisting symptoms had significantly more problems at work and for those with ADHD symptoms lessened in adulthood, the negative impact of earlier ADHD symptoms can still be seen on occupational outcomes.

##### 3.3.3.3 Quality of life

ADHD has been found to impair the quality of life in adults (172) and children (173). ADHD has a comparable overall impact on QoL compared to other mental health conditions and severe physical disorders, with increased symptom levels and impairment predicting poorer QoL (173). While robust negative effects on QoL are reported by the parents of children with ADHD across a range of psycho-social, achievement and self-evaluation domains, children with ADHD rate their own QoL less negatively than their parents and do not always see themselves as functioning less well than healthy controls (173, 174). Children’s health-related QoL was also significantly poorer (174) with a moderate impact on physical health-related QoL but a large impact on psychosocial domains (175, 176). Children with attention deficit hyperactivity disorder had more problems in all psychosocial domains and family activities, including mental health, self-esteem, parental impact, and emotional/behavioural (176).

##### 3.3.3.4 Relationships and social interactions

ADHD is also linked with relationship and social difficulties spanning a wide range of impacts (177–181).

Children with ADHD have significantly more impairment in peer functioning and social skills than non-ADHD peers (178). Social dysfunction and peer interaction problems are especially salient in girls with ADHD, who demonstrate increased difficulties in domains of friendship, peer interaction, social skills and functioning, and peer victimization (179). Social functioning difficulties also impact children’s interactions with teachers. Students with ADHD indicate feeling less close to their teacher while teachers experience less emotional closeness, less cooperation and more conflicts with their students with ADHD compared to other students (181).

Additionally, ADHD strongly impacts intimate relationships in adults. Adults with ADHD are more likely to divorce and report less satisfaction and more trouble navigating romantic relationships, and are more likely to have less intimacy and fear of intimacy (180). ADHD is also associated with intimate partner violence (IPV), anger, hostile conflict, and low conflict resolution (180). In terms of sexual relationships, Soldati and colleagues (177) demonstrated that individuals with ADHD report less sexual satisfaction, more sexual desire, more masturbation frequency, more sexual dysfunctions, poorer sexual health and difficulty in romantic relationships. Finally, difficulties with parenting are also often observed in adults with ADHD (180, 182).

##### 3.3.3.5 Risk-taking

Five papers explored risk-taking behaviour, three related to driving (55, 158, 183), one pregnancy (182) and one general risk-taking (184).

The three reviews on ADHD and driving all revealed an overall increased risk of accidents in people with ADHD. Jerome (158) found a relative risk ratio of 1.54, however, a meta-analysis conducted by Vaa (55) found a lower risk of 1.29 (1.12; 1.49) when correcting for publication bias, and 1.23 (1.04; 1.46) when adjusting for exposure. Vaa (55) also found that whereas ADHD drivers have more speeding violations, they do not have more drunk or reckless driving citations than drivers without ADHD. Similarly, Jerome (159) also noted that the relationship between ADHD and driving incidents was more evident in violations and citations rather than vehicle crashes. Deshmukh (183) found ADHD led to more traffic citations, accidents and licence suspension and postulated that this may be a result of road rage exacerbated by ADHD characteristics.

The review that explored the relationship between ADHD and pregnancy found that ADHD was associated with an increased risk of teenage pregnancy (182). Maternal ADHD was also associated with pregnancy complications (including eclampsia, infection, and caesarean section). The review found no increased risk of malformations because of ADHD treatment during pregnancy. In fact, there was some evidence that stimulant treatment during pregnancy was associated with a lower risk for pregnancy and birth complications (e.g. miscarriage, and placental dysfunctions).

Finally, one meta-analysis looked at overall risk-taking by reviewing data from behavioural task studies, self-reports and virtual reality studies (184). The review found children and adults with ADHD show moderately greater risk-taking than those without ADHD. Sub-optimal decision-making was the only moderator, which was not impacted by age, gender, sub-type or co-morbid disruptive behaviour disorder.

##### 3.3.3.6 Other domains

Two reviews did not fit any of the criteria above. Thrower et al. (185) conducted a review exploring the relationship between Gender Dysphoria and ADHD. Their review found four papers (two in adult populations and two in child populations) and indicated a higher prevalence of ADHD in people with gender dysphoria. However, no studies explored gender dysphoria in ADHD specifically and papers were of low quality (e.g. retrospective clinical case review). Finally, Werling (186) revealed that youth with ADHD spend more time on digital media and have more severe symptoms of problematic internet.

## 4 Discussion

This umbrella review was conducted to establish the relationships between ADHD and a range of potentially adverse outcomes in the domains of physical and mental health, and social and lifestyle functioning. It aimed to identify systematic associations based on previous reviews and meta-analyses to provide a comprehensive picture of outcomes related to ADHD. The findings revealed that the outcomes most commonly and consistently associated with ADHD were addiction, other mental health disorders, sleep disorders, overweight/obesity, accidents/injuries, offending and criminality, lower educational attainment/occupational functioning, reduced quality of life, relationship difficulties, and risky behaviours such as driving accidents/convictions and unplanned pregnancy. These findings have implications for improved identification efforts and developing effective interventions and support for those living with ADHD to help mitigate the impact of adverse outcomes on their health and wellbeing. We now explore the main findings in greater depth and their relevance to the management of ADHD and to policy development to improve support for people with ADHD.

### 4.1 Mental health

Mental health outcomes including addiction, self-harm and suicidality, psychiatric and personality disorders, and poor self-esteem were strongly and consistently associated with ADHD across most reviews included in this synthesis. The reviews we rated as being of ‘good’ quality often included over 50 studies within their analysis, demonstrating the strength of evidence in this area. In the area of addiction, there was consistent evidence that ADHD is associated with addictions to recreational drugs, alcohol and nicotine. For example, the review of Charach et al. (73) reported a significant association between childhood ADHD and alcohol use in adulthood and nicotine in adolescence, although the evidence of an association with drug use was less clear. This review included 49 studies and used appropriate methods of data extraction, synthesis and analysis, providing one of the strongest pieces of evidence in support of an association between ADHD diagnosis in childhood and substance use disorders later in development. Newer areas of research which included fewer reviews showed evidence of associations between ADHD and internet, gaming and gambling addictions, suggesting a relationship between ADHD and addiction which transcends a range of types of addiction. Further research is needed to disentangle the links between ADHD and addiction. For instance, impulsivity, particularly a preference for novelty and sensation-seeking, are core features of ADHD and are also strongly implicated in addictive behaviours (187, 188) making it difficult to clarify which issue comes first in the developmental trajectory. Treatment with methylphenidate is associated with a reduced risk of substance misuse (189) suggesting that reducing ADHD symptoms leads to better outcomes in this area. Arguably, ADHD medications could also reduce addictive behaviours via primary effects on dopamine and norepinephrine (190). Further research is therefore needed to disentangle the complex interactions between ADHD and addiction. Nonetheless, the findings of this review highlight the importance of strategies to mitigate the risk of addictive behaviours in ADHD.

Other aspects of mental health that were associated with ADHD include increased suicidal behaviours and mood disorders, particularly bipolar disorder, in addition to personality disorder. The findings were highly consistent with 8/8 reviews showing a positive association between ADHD and risk of suicide/self-harm, and 8/8 reviews showing increased risk of mental health disorders. Of these, several reviews were rated as poor or moderate quality, undermining the weight of evidence in this area. However, the reviews rated as good quality indicate that these associations are worthy of consideration when developing healthcare strategies for ADHD. In particular, Septier (52) reviewed a large number of studies (59) and used reliable methods for selection and synthesis across studies. They report a significant association between ADHD and suicidal attempts, ideations, plans and completed suicide, highlighting that the ADHD population should potentially be considered as an at-risk group for mental health professionals carrying out risk assessments around self-harm or suicidal intent. Similarly, the review of Allely et al. (29) showed increased rates of self-harm in ADHD.

These findings suggest some commonality in the pathophysiology of these conditions, and this is supported by previous research. One area of potential overlap is emotional dysregulation which is common to ADHD and to mood and personality disorders. Similarly, impulsivity is a core feature of ADHD and may also play a role in completed suicide and self-harm (191). Poor mental health is an important and consistent adverse outcome of ADHD, but it is not always easy to differentiate between ADHD and the clinical profile of these other diagnostic categories. Further research is therefore needed to establish whether these outcomes are latent comorbidities which only emerge at specific life stages, or whether ADHD is a precursor to and risk factor for these adverse outcomes which has implications for treatment and support. For instance, effective treatment of ADHD symptoms may be the best approach to reduce the risk of the onset of an additional mental health disorder, but additional or alternative treatment may be required if this is arising as a comorbidity instead. Furthermore, the poor self-esteem consistently reported in the reviews may be linked closely to mood disorders given that it is often associated with depression.

In summary, adverse mental health outcomes are clearly associated with ADHD and these may require specific intervention to mitigate their onset and improve prognosis. Tailored support is needed for effective treatment of mental health difficulties among people with ADHD as some existing interventions may not be effective given their symptoms and interacting challenges.

### 4.2 Physical health

Physical health outcomes including sleep, oral health, weight, accidents and injuries, and disease, were also strongly and consistently associated with ADHD across most reviews included in this synthesis. Unlike with mental health, there was a less oblivious relationship between the number of included studies and the overall quality of the review.

Thirteen reviews contributed to our conclusions on sleep, covering over 300 studies. An early review (108) which was rated as poor on our quality assessment suggested significant differences in movement in sleep and sleep apnea, but no differences in sleep-onset or bedtime resistance between ADHD children and controls. However subsequent reviews (109) highlighted parental reports of challenges around bedtime and in the early morning. The limited number of methodologically sound studies in this area was highlighted, which is disappointing as there are objective measures of sleep that can be collected via actigraphy, unlike in mental health where outcomes are nearly always restricted to self or parental report. Greater levels of period limb movement have also been shown to be more prominent in children with ADHD compared to controls (117) although it is not clear how periodic limb movement was differentiated from fidgeting by raters in those studies. Similar results were found for Adults with ADHD, but the bi-directional relationship between poor sleep and ADHD symptoms was much clearer in adult studies. This underlines both the impact of Sleep on ADHD symptom expression, but also how it may create a vicious cycle that maintains both poor sleep and high symptom expression for adults, exacerbating impairments. A high-quality review (113) that explored sleep and ADHD over the lifespan, showed a clear association between lower sleep levels and higher ADHD symptoms. It is tantalising to speculate how improved sleep could reduce Adult ADHD symptom expression (e.g. inattention) and reduce impairment and more support for sleep problems in children and adults with ADHD could provide a credible indirect route for ADHD symptom and impairment reduction.

Six reviews focussed on oral health all were rated good in our quality assessment. Reviews showed that children with ADHD were at a greater risk of dental problems including higher levels of tooth decay and tooth cavities, higher plaque scores and higher dental trauma risks. The mechanisms that might underscore differences in oral hygiene are unclear, but being impatient and impulsive may lead to less time spent brushing, inattention may lead to more frequent skipping of teeth brushing, and the link between ADHD and obesity may also suggest that children with ADHD eat more sugary foods. Previously discussed sleep difficulties in children with ADHD may also play a role in oral hygiene. Bedtime resistance and difficulties establishing and maintaining daily routines may encourage parents to also skip tooth brushing when they finally manage to get their child into bed, and poor sleep quality may explain the higher occurrence of tooth grinding in children with ADHD.

Nine reviews explored the relationship between weight and ADHD although studies were of variable quality. Results consistently demonstrated a significant association between ADHD and obesity, measured in different ways including overall weight and BMI, findings which parents and clinicians may find surprising, given the stereotype of an ADHD child as being full of energy and never sitting still. This association appears to be universal, with Li and colleagues (131) showing especially high levels of obesity in children with ADHD in Europe and Asia. This association between ADHD and obesity can also be mediated by poverty: poverty is often linked with parental ADHD and thus to poorer nutrition. A greater understanding of how to combat obesity in children with ADHD is required, including the potential moderating role of stimulant medication, as well as a greater understanding of how inattention and impulsivity may influence eating patterns.

Eight reviews explored accidents, injuries and ADHD, all of moderate quality. Reviews consistently demonstrated the link between ADHD and a higher risk of accidents, including poisoning, non-intentional injury and bone fractures. The risk of greater accidents was present in both children and adults with ADHD, with a greater risk of traumatic brain injury in adults that was not present in childhood. This risk for adults is most likely associated with the much higher level of car and motorbike accidents experienced by adults with ADHD. Finally, while not always accidental, the link between ADHD and unnatural death is much higher than expected.

Thirteen reviews highlighted the link between ADHD and disease and impairment, studies were all moderate to good in terms of quality. ADHD was associated with a range of diseases or impairing experiences, including asthma, migraine, chronic pain, and sight problems. Evidence for the link between ADHD and celiac disease was inconclusive with different reviews finding evidence for and against an association. While disparate in nature, these findings do highlight the additional burden and impairments for individuals with ADHD over and above the direct burden of the disorder.

### 4.3 Lifestyle and social factors

There was consistent and strong evidence of associations between ADHD and criminal behaviour, poor educational attainment, and poor relationships, as well as increased levels of risk-taking behaviours including unplanned and teenage pregnancy, and driving-related incidents and offences. These associations were significant for all the reviews included in this umbrella review. Quality of life was also found to be significantly adversely impacted by ADHD in all the reviews included here.

Regarding criminal behaviours, offending and incarceration, there was clear evidence of an association. Mohr-Jensen et al. (163) conducted a large systematic meta-analysis of data gathered from registries held in 6 countries yielding a sample size of over 15,000 children and adolescents who were followed up into adulthood. They reported significantly elevated rates of convictions and imprisonment in ADHD and showed that offending behaviours had an earlier age of onset in ADHD compared with neurotypical peers. They also defined the types of criminal activity to have an impulsive component, indicating a link to one of the diagnostic features of ADHD, which is also linked to emotional dysregulation poor inhibitory control, and alcohol/substance misuse. Similarly, another small but high-quality review (159) reported a significant association between ADHD and intimate partner violence, with some studies identifying alcohol use and comorbid conditions including conduct disorder (CD) and antisocial personality disorder (ASPD) as mediators. It is important to note that one of the reviews (160) found that ADHD is also associated with an increased risk of being a victim of intimate partner and sexual violence. This suggests that the relationships between ADHD and domestic or intimate partner violence are complex and multifactorial, with some of the association explained by additional factors. Some studies included in these reviews suggest that impulsivity may be a core underpinning construct explaining increased criminal activity and violence, and this would also explain the transdiagnostic associations with CD, ASPD, and alcohol use highlighted by Buitelaar et al. (161), which need further exploration. Overall, the evidence of significantly increased prevalence of ADHD in prison settings confirms that this is a notable area of poor outcomes in ADHD and should therefore be a focus of further research and policy development to reduce the likelihood of offending in those with an ADHD diagnosis, particularly when there is comorbidity with CD, ASPD and alcohol use disorder.

Poor educational attainment and occupational outcomes are also significantly associated with ADHD, particularly when ADHD is untreated, and these associations remain significant when variation in intellectual ability is accounted for. Our analysis found worse outcomes from all 5 reviews in this domain which synthesised data from over 200 studies. The outcomes synthesised include leaving school early, achieving lower academic qualifications, unemployment and frequent job changes, and lower pay compared with peers. Christiansen et al. (170) also noted that those with more severe symptoms persisting into adulthood fared worse in employment. The evidence in this domain indicates that poorer academic outcomes are likely to lead to poor employment experiences, highlighting an urgent need to cater for children with ADHD in the classroom to scaffold their learning more effectively, engage them in academic activities more fully, and support them into further studies/employment post-education. Under the Equality Act in the UK (192), ADHD is classified as a disability for which employers must make reasonable adjustments. There is compelling evidence describing the challenges of ADHD symptoms in the workplace (5) but very little research into effective workplace interventions that can support ADHD in the workplace (193). Further research is urgently needed to investigate how best to support children with ADHD in schools, and adults with ADHD in the workplace/higher education institutions. This is particularly important given the increase in adults presenting to services requesting a diagnostic assessment; the ultimate outcome of such an assessment must include recommendations for educators and employers.

Five reviews were included which explored associations between ADHD and relationship difficulties. Between them, these reviews included over 100 studies in this area, designed to assess a range of indicators of social functioning, peer relationships and intimate relationships. All reported evidence of poorer functioning in individuals with ADHD. This manifests as difficulties forming and maintaining peer and intimate relationships, poorer social skills, and differences in social cognition, which may be partially mediated by CD in children (178). Previous research has shown an overlap between ADHD and CD in explaining social cognitive differences in children with ADHD (194). Furthermore, ADHD and autism spectrum disorders frequently co-occur and there is evidence of shared genetic and phenotypic aetiology (195, 196), including difficulties in core social processing skills such as following eye gaze (197, 198). Despite this, none of the reviews included here mentioned autism as a possible mediating factor in explaining relationship difficulties in ADHD, highlighting an area for further research.

Of six reviews focusing on risk-taking behaviours in ADHD, 4 focussed on driving and showed evidence of significantly increased rates of a range of driving outcomes, including accidents, collisions, road rage, and driving citations and offences. The methods of these systematic reviews were judged to be poor quality, largely due to a lack of detail in the methods around core aspects of the search strategy such as the number of databases searched, lack of reliability checks for screening and data extraction, and no quality rating of the included papers. Two of the reviews also overlapped considerably in their included papers and findings. Notably, Vaa (55) highlighted a potential role for CD and ODD in explaining the link between ADHD and driving-related outcomes, suggesting that previous research had overlooked these mediators, and they should be taken into consideration in future research. Another review in this domain focussed on ADHD and pregnancy (182) and reported significant associations between childhood ADHD and CD symptoms and unplanned and teenage pregnancies, with lower academic achievement and substance misuse associated with risky sexual behaviours in girls with ADHD. The authors highlight the lack of research investigating boys with ADHD and predictors of unplanned parenthood at an early age. This area needs further research given the potential impacts on other life outcomes for the individual and the costs to health and social care from unplanned and teenage pregnancies.

Research investigating ADHD has tended to focus on symptom ratings as the primary outcome. Recently, there has been an increased emphasis on other outcomes that may ultimately have greater relevance to those living with ADHD, including quality of life (199). All five reviews included in this umbrella review demonstrated a significant association between ADHD and reduced quality of life, particularly in the areas of school and psychosocial functioning and family and social relationships, with less clear findings related to physical functioning. This may be partly a consequence of the measures used in this field of research which have often been designed for populations with physical rather than mental health difficulties. The reviews also found that parent-rated QoL tended to be lower than children’s ratings where both were gathered. This is a common feature of ADHD, that children (particularly those younger than 12-15 years) may be less able to reflect on or describe their own difficulties and challenges, or they may perceive certain aspects of ADHD symptoms (e.g. impulsiveness) positively, and this has led to a reliance on proxy reports (teachers, parents, school observations) for evaluating children with ADHD. Another interpretation advanced by Danckaerts et al. (173) is that parent ratings of their children’s QoL reflect their burden as parents, and that further work is needed to understand in more depth children’s perspectives on their QoL.

### 4.4 Strengths and limitations

This is the first umbrella review to explore the main impacts associated with having ADHD, including, physical, mental and lifestyle aspects. This review enables findings of reviews relevant to the review question to be compared and contrasted. As such, this review provides a valuable resource for clinicians and academics where these impacts are presented together and demonstrate the wide implications of ADHD. With over 1,000 studies included, this review has the unique advantage of incorporating years of research into one publication. The strengths of this review include the broad focus of the review and the thorough approach to selecting reviews for inclusion. The focus was deliberately broad in order to provide a comprehensive analysis of outcomes associated with ADHD in the core domains that have often been reported as negatively impacted by this condition, including mental health, physical health and lifestyle/social factors. This review enabled us to identify a variability of risks and impacts which can then guide future research studies to explore in greater depth the mechanisms of negative outcomes in ADHD and guide policy development to enhance a range of outcomes for those affected by ADHD.

Despite the strength of this approach, it is important to note that the findings of this review are inevitably limited by the quality of the included reviews and their underpinning studies. Many reviews were rated as poor or moderate, largely because the conduct or reporting of the methodology was unsatisfactory. The quality rating enabled us to determine a high variability in the quality of reviews, however, it is important to bear in mind that these are a reflection of the review methodologies rather than the individual studies and therefore limit our abilities to gauge the quality of each included studies. Our findings are thus constrained by the quantity, quality and comprehensiveness of the available information in the primary reviews. There was large heterogeneity in methodologies and statistical techniques across the reviews, as such, it was not possible to conduct a meta-analysis or directly compare findings between publications. The topics covered by this review are also limited due to the nature of umbrella reviews. While it would be impossible to look at every study investigating this important topic, a review of reviews was the most pragmatic and feasible approach to get a broad understanding. However, topics that have not been synthesised through a review will not have been captured and therefore important factors might be missing. This review was limited to papers published in the English language, resulting in nine studies being excluded. While it is not uncommon to restrict reviews to the English language, these papers might have added important nuances to this review. Finally, while most sub-themes included multiple reviews, a couple only included one. Self-esteem and gender dysphoria were only explored by one review, limiting the evidence for this topic.

### 4.4 Recommendations

Our findings highlight the importance of clinicians taking a holistic approach to the assessment and management of ADHD, being mindful of common co-occurring conditions and impacts on lifestyle. Many people and professionals are impacted by these findings, including healthcare practitioners, social, forensic, education and industries. In relation to mental health conditions, our review highlights the need for clinicians to be mindful in assessing for co-occurring difficulties in addiction, suicide, eating disorders, mood and personality disorders. Our review also indicates that clinicians should be aware of physical conditions such as sleep, oral hygiene, injury and obesity in relation to individuals with ADHD. Future research could explore the development of psychoeducation packages for families and adults to support these areas. These findings could also support the development of future tailored prevention or treatment interventions aimed specifically at ADHD populations, such as tailored exercise or diet management programmes to reduce obesity. Future research could also explore the under-represented areas of gender dysphoria and self-esteem.

## 5 Conclusion

In conclusion, this comprehensive review sheds light on the multifaceted impacts of ADHD, extending beyond its core symptoms and impairments. The findings reveal a spectrum of health and lifestyle risks associated with ADHD, encompassing mental health vulnerabilities such as addiction, suicide, eating disorders, mood, and personality disorders. Moreover, the review underscores the significance of recognising key physical health risks, notably obesity, sleep issues, oral hygiene, injuries, and somatic diseases.

Crucially, the review unveils the broader implications on lifestyle, encompassing areas such as offending behaviour, criminality, violence, employment, education, quality of life, relationships, and risk-taking. This holistic perspective emphasises the interconnectedness of ADHD with various aspects of an individual’s life and societal dynamics.

This research is the first to systematically illuminate the extensive ramifications of ADHD. The identified impacts underscore the necessity of adopting a holistic approach in the realms of recognition, treatment, research, and support for individuals with ADHD. As we move forward, it is imperative to integrate this comprehensive understanding into the discourse surrounding ADHD, fostering a more nuanced and effective approach to address the diverse challenges posed by this neurodevelopmental disorder. By doing so, we can enhance the well-being of individuals affected by ADHD and contribute to the development of more targeted interventions that consider the intricate interplay between the disorder and various aspects of life.

## Author contributions

BF: Conceptualization, Formal analysis, Investigation, Methodology, Writing – original draft, Writing – review & editing. GN: Data curation, Methodology, Software, Writing – review & editing. HW: Investigation, Project administration, Resources, Validation, Writing – review & editing. KS: Conceptualization, Supervision, Writing – review & editing. DD: Conceptualization, Investigation, Methodology, Supervision, Visualization, Writing – review & editing. MG: Conceptualization, Methodology, Project administration, Supervision, Writing – review & editing. SC: Conceptualization, Investigation, Supervision, Writing – review & editing. CH: Formal Analysis, Investigation, Methodology, Visualization, Writing – original draft, Writing – review & editing.
