# Supplementary material for: The impacts associated with having ADHD: an umbrella review
Source: Front Psychiatry. 2024 May 21;15:1343314. doi: 10.3389/fpsyt.2024.1343314 (PMC11151783; doi:10.3389/fpsyt.2024.1343314)
Supplement: Supplementary file 2 [file Table_1.docx]

# Table 1 - Included studies

| Mental health | | | | | | | | | | | | | | | | | | |
| --- | --- | --- | --- | --- | --- | --- | --- | --- | --- | --- | --- | --- | --- | --- | --- | --- | --- | --- |
| First author, year | Title of review | | Year of studies (range) | | Topic comparison | | | Population of interest | | Number of studies included | | Type of review | | Synopsis of findings | | Quality assessment score | Quality assessment rating | |
| Addiction | | | | | | | | | | | | | | | | | | |
| Bing-qian, 2017 | The association between attention deficit/hyperactivity disorder and internet addiction: A systematic review and meta-analysis. | | 2007-2016 | | Internet addiction | | | Children and adults | | 15 | | Systematic review and meta-analysis | | This review shows that there is a link between ADHD and internet addiction. | | 1 | Good | |
| Charach, 2011 | Childhood attention-deficit/hyperactivity disorder and future substance use disorders: Comparative meta-analyses | | 1984-2008 | | Substance abuse/addiction | | | Children and adults age range (13.6-25) | | 13 | | Systematic review and meta-analysis | | Children with ADHD are at a higher risk of developing alcohol use disorder, nonalcohol drug use disorders and nicotine use | | 0.86 | Good | |
| Dullur, 2021 | A systematic review on the intersection of attention-deficit hyperactivity disorder and gaming disorder | | 2006-2019 | | Gaming disorder | | | Children and adults (age range 4.4-88y) | | 29 | | Systematic review | | The review shows an association between ADHD and gaming disorder although the direction of the relationship is unclear | | 0.65 | Moderate | |
| Gonzalez-Bueso, 2018 | Association between Internet Gaming Disorder or Pathological Video-Game Use and Comorbid Psychopathology: A Comprehensive Review. | | 2011-2018 | | Gaming disorder | | | Children and adults | | 24 | | Systematic review | | The review shows a correlation between ADHD and gaming disorder. | | 0.55 | Moderate | |
| Ho, 2014 | The association between internet addiction and psychiatric co-morbidity: A meta-analysis | | 2004-2009 | | Internet addiction | | | Children and adolescents | | 4 | | Systematic review and meta-analysis | | This review shows a link between internet addiction and ADHD | | 0.68 | Moderate | |
| Karaca, 2017 | Comorbidity between Behavioural Addictions and Attention Deficit/Hyperactivity Disorder: a Systematic Review | | 1995-2015 | | Addiction | | | Children and adults | | 14 | | Systematic review | | This systematic review showed a high degree of co-occurrence of ADHD and behavioural addictions | | 0.4 | Poor | |
| Lee, 2011 | Prospective association of childhood attention-deficit/hyperactivity disorder (ADHD) and substance use and abuse/dependence: a meta-analytic review. | | 1984-2008 | | Substance abuse/addiction | | | Children and adults | | 27 | | Systematic review and meta-analysis | | The review shows that ADHD is linked with substance use disorder and dependence. | | 0.55 | Moderate | |
| Luderer, 2021 | Alcohol use disorders and ADHD. | | Not reported (NR) | | Alcohol use disorder (AUD) | | | Adults and adolescents | | NR | | Literature review | | This review shows a significant ADHD comorbidity in substance-abusing populations | | 0.67 | Moderate* | |
| Martinez-Raga, 2013 | Attention deficit hyperactivity disorder and dual disorders. Educational needs for an underdiagnosed condition. | | NR | | Substance use disorder (SUD) | | | Children and adults | | 49 | | Literature review | | The review shows that there is a link between ADHD and SUD. | | 0.75 | Good* | |
| Oliva, 2021 | Prevalence of cocaine use and cocaine use disorder among adult patients with attention-deficit/hyperactivity disorder: A systematic review and meta-analysis. | | 1995-2016 | | SUD | | | Adults | | 12 | | Systematic review and meta-analysis | | This review shows that there is a link between ADHD, cocaine use and cocaine use disorder with a high prevalence of cocaine use in adults with ADHD | | 0.64 | Moderate | |
| Rohner, 2023 | Prevalence of Attention Deficit Hyperactivity Disorder (ADHD) among Substance Use Disorder (SUD) Populations: Meta-Analysis | | 1989-2009 | | SUD | | | Children and adults | | 29 | | Meta-analysis | | This review shows that ADHD is linked with SUD | | 0.68 | Moderate | |
| Salerno, 2022 | ADHD-Gaming Disorder Comorbidity in Children and Adolescents: A Narrative Review | | 2012-2022 | | Addiction/gaming | | | Children and adults | | 20 | | Narrative review | | Yes, this review shows that there is a link between ADHD and gaming, ADHD being risk factors for problematic gaming | | 0.75 | Good* | |
| Theule, 2019 | Exploring the relationships between problem gambling and ADHD: A meta-analysis | | 1992-2014 | | Gambling | | | Children and adults | | 24 | | Meta-analysis | | This review shows a significant correlation between ADHD and gambling | | 0.64 | Moderate | |
| Van Emmerik-van, 2012 | Prevalence of attention-deficit hyperactivity disorder in substance use disorder patients: a meta-analysis and meta-regression analysis | | NR | | SUD | | | Adolescents and adults | | 29 | | Meta-analysis | | This review shows that a high proportion of SUD subjects also have ADHD | | 0.41 | Poor | |
| Weinstein, 2012 | Emerging association between addictive gaming and attention-deficit/hyperactivity disorder | | NR (searched from 2000 to 2012) | | Gaming disorder/addiction | | | Children and adults | | NR | | Literature review | | The review shows evidence of an association between computer game and videogame addiction and ADHD | | 0.67 | Moderate* | |
| Wilens, 2006 | Alcohol, drugs, and attention-deficit/hyperactivity disorder: A model for the study of addictions in youth | | NR | | Addiction | | | Children and adults | | NR | | Literature review | | ADHD increases the risk for cigarette smoking and SUD and is associated with greater SUD severity | | 0.67 | Moderate* | |
| Suicide and self harm | | | | | | | | | | | | | | | | | | |
| Garas, 2020 | Long-Term Suicide Risk of Children and Adolescents With Attention Deficit and Hyperactivity Disorder-A Systematic Review | | 2008-2020 | | Suicide | | | Children and adults (age range: from birth to 40) | | 18 | | Systematic review | | This review shows a link between ADHD and suicidal thoughts and attempts. | | 0.83 | Good | |
| James, 2004 | Attention deficit hyperactivity disorder and suicide: A review of possible associations | | NR | | Suicide | | | Children and adults | | 22 | | Literature Review | | The review found an association of ADHD and completed suicide, especially for younger males | | 0.5 | Moderate* | |
| Giupponi, 2018 | Suicide risk in attention-deficit/hyperactivity disorder. | | 1990-2017 | | Suicide | | | Children and adults | | NR | | Literature review | | The review shows a link between ADHD and suicidal behaviour. | | 0.83 | Moderate* | |
| Septier, 2019 | Association between suicidal spectrum behaviors and Attention-Deficit/Hyperactivity Disorder: A systematic review and meta-analysis | | 2001-2018 | | Suicide | | | Children and adults | | 57 | | Systematic review and meta-analysis | | This review shows a link between ADHD and suicidal attempts, suicidal ideations, suicidal plans, and suicide | | 0.82 | Good | |
| Balazs, 2017 | Attention-deficit/hyperactivity disorder and suicide: a systematic review. | | 2011 to 2015 | | Suicide | | | Children and adults | | 26 | | Systematic review | | The review shows that there is a link between ADHD and suicidality in all age groups and both sexes. | | 0.55 | Moderate | |
| Impey, 2012 | Completed suicide, ideation and attempt in attention deficit hyperactivity disorder | | NR | | Suicide | | | Children and adults | | NR | | Literature review | | This review demonstrates a positive association between ADHD and completed suicide, attempt and ideation | | 0.5 | Moderate* | |
| Furczyk, 2014 | Adult ADHD and suicide | | NR | | Suicide | | | Children and adults | | NR | | Literature review | | Individuals with ADHD are at an increased suicide risk. | | 0.58 | Moderate* | |
| Allely, 2014 | The association of ADHD symptoms to self-harm behaviours: a systematic PRISMA review. | | 1997-2012 | | Self-harm | | | Children and adults | | 15 | | Systematic review | | The review shows an association between ADHD and self-harm. | | 0.7 | Good | |
| Mood and personality disorders | | | | | | | | | | | | | | | | | | |
| Abramovitch, 2015 | Comorbidity between Attention Deficit/Hyperactivity Disorder and Obsessive-Compulsive Disorder Across the Lifespan: A Systematic and Critical Review. | | 1990-2013 | | Obsessive-compulsive disorder (OCD) | | | Children (mean age range 2.3-18 years) | | 48 | | Systematic and Critical Review | | This review shows that ADHD is linked with OCD in children but not in adults. | | 0.6 | Moderate | |
| Biederman, 1991 | Comorbidity of attention deficit hyperactivity disorder | | 1979-1991 | | Conduct, Depressive, Anxiety, and Other Disorders | | | Children and adults | | 29 | | Literature review | | The review shows a link between ADHD and conduct disorder, oppositional defiant disorder, mood disorders, anxiety disorders, learning disabilities, and borderline personality disorder. | | 0.67 | Moderate* | |
| Brancati, 2021 | Development of bipolar disorder in patients with attention-deficit/hyperactivity disorder: A systematic review and meta-analysis of prospective studies | | 2006-2021 | | Bipolar disorder (BD) | | | Children (age range 6-18 years) | | 10 | | Systematic review and meta-analysis | | This review shows that ADHD is linked with the co-occurrence of bipolar disorder. | | 0.64 | Moderate | |
| Davids, 2005 | Attention deficit hyperactivity disorder and borderline personality disorder. | | NR | | Borderline personality disorder (BPD) | | | Adults | | NR | | Literature review | | This review shows that ADHD is associated with BPD | | 0.5 | Moderate* | |
| Faraone, 2012 | Examining the comorbidity between attention deficit hyperactivity disorder and bipolar I disorder: a meta-analysis of family genetic studies | |  | | Bipolar I disorder | | | Children (age range 5-12 years) | | 27 | | Systematic review and meta-analysis | | This review found a significantly higher prevalence of ADHD among relatives of bipolar I and a significantly higher prevalence of bipolar I disorder among relatives of ADHD. | | 0.5 | Moderate | |
| Meinzer, 2014 | The co-occurrence of attention-deficit/hyperactivity disorder and unipolar depression in children and adolescents: a meta-analytic review | | 1987-2010 | | Unipolar depression | | | Children and adults | | 29 | | Meta-analysis | | Evidence of an association between ADHD and depression is mixed with a medium sized association between ADHD and depression | | 0.5 | Moderate | |
| Sandstrom, 2021 | Prevalence of attention-deficit/hyperactivity disorder in people with mood disorders: A systematic review and meta-analysis | | 1996-2020 | | BD and major depressive disorder (MDD) | | | Children and adults | | 127 | | Systematic review and meta-analysis | | This review shows that ADHD is common in individuals with BD and MDD | | 0.59 | Moderate | |
| Schiweck, 2021 | Comorbidity of ADHD and adult bipolar disorder: A systematic review and meta-analysis | | 1993-2020 | | BD | | | Adults | | 80 | | Systematic review and meta-analysis | | This review demonstrates that ADHD and BD highly co-occur | | 0.77 | Good | |
| Skirrow, 2012 | An update on the debated association between ADHD and bipolar disorder across the lifespan | | 2005-2011 | | BD | | | Children and adults) | | 17 | | Literature review | | The review shows that there is a link between ADHD and bipolar disorder | | 0.67 | Moderate* | |
| Zdanowicz, 2010 | ADHD and bipolar disorder among adolescents: nosology in question. | | NR | | BD | | | Children and adults | | NR | | Literature review | | This review shows a bidirectional association between ADHD and BD | | 0.58 | Moderate* | |
| Other disorders | | | | | | | | | | | | | | | | | | |
| Arican, 2018 | The prevalence of attention deficit hyperactivity disorder symptoms in a cohort of patients with schizophrenia, and the impact on their executive functions | | 2009-2014 | | Schizophrenia | | | Adults | | 5 | | Systematic review | | The review shows a link between schizophrenia and ADHD. | | 0.4 | Poor | |
| Choi, 2022 | The prevalence of psychiatric comorbidities in adult ADHD compared with non-ADHD populations: A systematic literature review. | | 2006-2022 | | Psychiatric comorbidities | | | Children and adults | | 32 | | Systematic literature review | | This review shows that there is a link between ADHD and psychiatric disorders. The most frequent comorbid psychiatric disorder was substance use disorder (SUD), followed by mood disorders, anxiety disorders, and personality disorders. | | 0.6 | Moderate | |
| Kaisari, 2017 | Attention Deficit Hyperactivity Disorder (ADHD) and disordered eating behaviour: A systematic review and a framework for future research. | | 1995-2016 | | Eating disorder (ED) | | | Children and adults | | 72 | | Systematic review | | This review shows that ADHD is linked with disordered eating and with specific types of disordered-eating behaviour, in particular, overeating behaviour. | | 0.95 | Good | |
| Levin, 2016 | Attention-deficit/hyperactivity disorder and eating disorders across the lifespan: A systematic review of the literature. | | 1995-2016 | | ED | | | Children and adults) | | 37 | | Systematic review | | This systematic review found evidence for an association between childhood ADHD and the later development of an ED or disordered eating | | 0.7 | Good | |
| Nazar, 2016 | The risk of eating disorders comorbid with attention-deficit/hyperactivity disorder: A systematic review and meta-analysis | | 2002-2016 | | ED | | | Children and adults | | 22 | | Systematic review and meta-analysis | | This review showed that there is a link between ADHD and having an ED. | | 0.77 | Good | |
| Nickel, 2019 | Systematic review: Overlap between eating, autism spectrum, and attention-deficit/hyperactivity disorder | | NR | | ED | | | Children and adults | | 25 | | Systematic review | | This review shows that studies that have focused on the prevalence of EDs in ADHD are heterogeneous, and they vary widely between no relationship between EDs and ADHD to significant | | 0.6 | Moderate | |
| Nourredine, 2021 | Association of Attention-Deficit/Hyperactivity Disorder in Childhood and Adolescence with the Risk of Subsequent Psychotic Disorder: A Systematic Review and Meta-analysis | | 2003-2020 | | Psychotic disorder | | | Children and adults | | 15 | | Systematic review and meta-analysis | | This review shows an increased risk of psychotic disorder for individuals with childhood ADHD | | 0.96 | Good | |
| Self esteem | | | | | | | | | | | | | | | | | | |
| Cook, 2014 | The self-esteem of adults diagnosed with attention-deficit/hyperactivity disorder (ADHD): A systematic review of the literature. | |  | | Self-esteem | | | Adults | | 13 | | Systematic review | | This reviewed showed that ADHD is associated with lower self-esteem in adulthood | | 0.86 | Good | |
| Physical Health | | | | | | | | | | | | | | | | | | |
| Sleep | | | | | | | | | | | | | | | | | | |
| Bondopadhyay, 2022 | | A Systematic Review of Sleep and Circadian Rhythms in Children with Attention Deficit Hyperactivity Disorder | | 2009-2018 | | Sleep | Children (age range birth to 13 years) | | 148 | | Systematic review | | This review shows that ADHD is linked with sleep disturbances in children. | | 0.83 | | | Good |
| Cortese, 2006 | | Sleep and alertness in children with attention-deficit/hyperactivity disorder: A systematic review of the literature. | | 1998-2004 | | Sleep | Children (mean age range 8.1-15.4) | | 5 | | Systematic review and meta-analysis | | This review shows that the children with ADHD are significantly more impaired than the controls in most of sleep measures | | 0.41 | | | Poor |
| Cortese, 2009 | | Sleep in Children with Attention-Deficit/Hyperactivity Disorder: Meta-Analysis of Subjective and Objective Studies | | 1987-2008 | | Sleep | Children (age range 5-12) | | 16 | | Systematic review and meta-analysis | | This review shows that children with ADHD are significantly more impaired than the controls in most of the subjective and some of the objective sleep measures. | | 0.64 | | | Moderate |
| Díaz-Román, 2018 | | Sleep in adults with ADHD: Systematic review and meta-analysis of subjective and objective studies. | | 2001-2017 | | Sleep | Adults | | 13 | | Systematic review and meta-analysis | | Yes, this review shows that subjective sleep problems are significantly associated with ADHD in adults. | | 0.91 | | | Good |
| Kim, 2020 | | Prevalence of attention deficit hyperactivity disorder symptoms in narcolepsy: a systematic review | | 2013-2018 | | Sleep (narcolepsy) | Children and adults | | 5 | | Systematic review and meta-analysis | | The review shows a link between ADHD and narcolepsy. | | 0.45 | | | Moderate |
| Lee, 2019 | | Association between sleep duration and attention-deficit hyperactivity disorder: A systematic review and meta-analysis of observational studies | | 2009-2018 | | Sleep | NR | | 10 | | Systematic review and meta-analysis | | The review shows that individuals with ADHD have shorter sleep duration. | | 0.82 | | | Good |
| Lugo, 2020 | | Sleep in adults with autism spectrum disorder and attention deficit/hyperactivity disorder: A systematic review and meta-analysis. | | 2005-2019 | | Sleep | Adults | | 28 | | Systematic review and meta-analysis | | This review shows that there is a link between ADHD and sleep disorder. | | 0.91 | | | Good |
| Lunsford-Avery, 2016 | | Sleep disturbances in adolescents with ADHD: A systematic review and framework for future research | | 2000-2015 | | Sleep disturbances | Children (age range 10-18 years) | | 25 | | Systematic review | | This review shows that ADHD is linked with more sleep disturbances | | 0.65 | | | Moderate |
| Martins, 2019 | | Sleep disturbance in children with attention-deficit hyperactivity disorder: A systematic review | | 2001-2017 | | Sleep | Children (age range: 7-12 years) | | 8 | | Systematic review | | This review demonstrates that children with ADHD have more sleep disturbance | | 0.9 | | | Good |
| Sadeh, 2006 | | Sleep in children with attention-deficit hyperactivity disorder: A meta-analysis of polysomnographic studies | | 1981-2005 | | Sleep | Children (age range 3.5-16 years) | | 12 | | Meta-analysis | | This review shows that children with ADHD are more likely to have sleep disorder. | | 0.5 | | | Moderate |
| Salerno, 2016 | | Sleep disorders in adult ADHD: A key feature | | NR | | Sleep | Adults | | 35 | | Literature review | | The review a link between ADHD and sleep disorders in adults | | 0.67 | | | Moderate* |
| Sedky, 2014 | | Attention deficit hyperactivity disorder and sleep disordered breathing in paediatric populations: a meta-analysis | | 2000-2012 | | Sleep disordered breathing | Children (age range 2-18 years) | | 18 | | Meta-analysis | | This review shows that sleep disordered breathing is more likely in children with ADHD | | 0.77 | | | Good |
| Youssef, 2011 | | Is obstructive sleep apnea associated with ADHD | | 1996-2006 | | Obstructive sleep apnea | NR | | 6 | | Literature review | | This review shows that ADHD is linked with obstructive sleep apnea. | | 0.67 | | | Moderate* |
| Oral health | | | | | | | | | | | | | | | | | | |
| Chau, 2020 | | Oral Health of Children With Attention Deficit Hyperactivity Disorder: Systematic Review and Meta-Analysis | | 2004-2013 | | Teeth | Children (Age range 30 months- 17.2) | | 26 | | Systematic review and meta-analysis | | This review shows that children with ADHD have poorer oral health. | | 0.91 | | | Good |
| Drumond, 2022 | | Periodontal outcomes of children and adolescents with attention deficit hyperactivity disorder: a systematic review and meta-analysis. | | 2008-2019 | | Teeth | Children (age range 6-17 years) | | 7 | | Systematic review and meta-analysis | | This review showed that there is a link between ADHD and gingival bleeding in children and adolescents. | | 0.86 | | | Good |
| Drumond, 2023 | | Dental trauma in children and adolescents with attention-deficit/hyperactivity disorder: A systematic review and meta-analysis. | | 2004-2020 | | Teeth | Children (age range:  1-18) | | 10 | | Systematic review and meta-analysis | | This review shows that children and adolescents with ADHD are more likely to have dental trauma than their non-ADHD peers. | | 1 | | | Good |
| Kammer, 2022 | | Prevalence of tooth grinding in children and adolescents with neurodevelopmental disorders: A systematic review and meta-analysis | | 1998-2019 | | Tooth grinding | Children | | 77 | | Systematic review and meta-analysis | | The review showed that tooth grinding was more common in children with ADHD | | 0.82 | | | Good |
| Manoharan, | | Dental Caries and Children  with Attention Deficit Hyperactivity Disorder (ADHD) - A Review | | NR | | Dental caries | Children | | NR | | Literature review | | This review does not show a link between ADHD and dental caries | | 0.75 | | | Good* |
| Souto-Souza,2020 | | Is there an association between attention deficit hyperactivity disorder in children and adolescents and the occurrence of bruxism? A systematic review and meta-analysis. | | 1998-2018 | | Bruxism | Children (age range 3-18) | | 27 | | Systematic review and meta-analysis | | The review shows a link between ADHD and bruxism (tooth grinding and clenching). | | 0.95 | | | Good |
| Weight | | | | | | | | | | | | | | | | | | |
| Cortese, 2008 | | Attention-deficit/hyperactivity disorder (ADHD) and obesity: a systematic review of the literature | | 1996-2007 | | Obesity | Children (age range 3-18 years) | | 10 | | Systematic review | | This review shows that there is a positive link between ADHD and obesity. | | 0.4 | | | Poor |
| Cortese, 2012 | | Obesity and ADHD: clinical and neurobiological implications. | | 1996-2009 | | Obesity | Children (age range 3-18 years) | | 18 | | Systematic review | | The review shows that children with ADHD are more likely to be obese. | | 0.3 | | | Poor |
| Cortese, 2016 | | Association Between ADHD and Obesity: A Systematic Review and Meta-Analysis. | | 2008-2014 | | Obesity | Children (age range 3-18 years) | | 42 | | Systematic review and meta-analysis | | The review shows that children with ADHD are more likely to be obese. | | 0.95 | | | Good |
| Cortese, 2017 | | Attention-Deficit/Hyperactivity Disorder (ADHD) and Obesity: Update 2016 | | 2013-2016 | | Obesity | Children (age range 5-18) | | 41 | | Review | | This review shows bidirectional relationship between ADHD and obesity. | | 0.7 | | | Good* |
| Guner, 2019 | | Comorbidity of attention deficit and hyperactivity disorder and obesity: A systematic review. | | 1996-2016 | | Obesity | Children (age range 3.5-18 years) | | 36 | | Systematic review | | The review shows that children with ADHD are more likely to be obese. | | 0.4 | | | Poor |
| Hanc, 2018 | | ADHD as a risk factor for obesity. Current state of research. | | 2004-2016 | | Obesity | Children and adults (age range 2-34) | | 31 | | Literature review | | This review shows that ADHD is linked with overweight and obesity. | | 0.67 | | | Moderate* |
| Li, 2020 | | Global prevalence of obesity, overweight and underweight in children, adolescents and adults with autism spectrum disorder, attention-deficit hyperactivity disorder: A systematic review and meta-analysis | | 2004-2020 | | Weight | Children (age range 6-12) | | 43 | | Systematic review and meta-analysis | | The review shows a high prevalence of obesity an overweight in children and adults with ADHD but not with underweight. | | 0.91 | | | Good |
| Nigg, 2016 | | Attention-deficit/hyperactivity disorder (ADHD) and being overweight/obesity: New data and meta-analysis | | 2006-2014 | | Obesity | Children (age range 3-18) | | 43 | | Systematic review and meta-analysis | | No, this review shows that there is no link between ADHD and obesity in children but showed a link in adulthood and possibly in adolescent girls | | 0.55 | | | Moderate |
| Zhu, 2023 | | Risk of Overweight and Obesity in Children and Adolescents with Attention-Deficit/Hyperactivity Disorder: A Systematic Review and Meta-Analysis | | 2011-2022 | | Weight | Children (age range 3-18) | | 16 | | Systematic review and meta-analysis | | This review showed a significant association between ADHD and overweight and obesity in both children and adolescents. | | 0.91 | | | Good |
| Injury | | | | | | | | | | | | | | | | | | |
| Adeyemo, (2014) | | Mild traumatic brain injury and ADHD: a systematic review of the literature and meta-analysis | | 1995-2009 | | Traumatic brain injury | Children and adults | | 5 | | Systematic review and meta-analysis | | The review shows that individuals with ADHD are more likely to experience Mild Traumatic Brain Injury | | 0.45 | | | Moderate |
| Amiri, 2017 | | Attention deficit/hyperactivity disorder and risk of injuries: A systematic review and meta-analysis | | 2003-2014 | | Injuries/ accidents | Children and adults | | 35 | | Systematic review and meta-analysis | | This review shows an increased risk of injury and ADHD. | | 0.82 | | | Good |
| Asarnow, 2021 | | Association of attention-deficit/hyperactivity disorder diagnoses with pediatric traumatic brain injury: A meta-analysis | | 1981-2020 | | Traumatic brain injury | Children (age range 4-18 years) | | 24 | | Systematic review and meta-analysis | | The review shows that there is a link between ADHD and Traumatic Brain Injury | | 0.64 | | | Moderate |
| Brunkhorst-Kanaan, 2021 | | ADHD and accidents over the life span - A systematic review | | 2003-2019 | | Accidents | Children and adults | | 82 | | Systematic review | | This review shows that ADHD is linked with an increased risk of accidents or unintentional injuries. | | 0.65 | | | Moderate |
| Catalá-López, (2022) | | Mortality in persons with autism spectrum disorder or attention-deficit/hyperactivity disorder: a systematic review and Meta-analysis. | | 1988-2019 | | Mortality | Children and adults | | 12 | | Systematic review and meta-analysis | | The review shows that ADHD is linked with increased risk of mortality | | 0.95 | | | Good |
| Ruiz-Goikoetxea, 2018 | | Risk of poisoning in children and adolescents with ADHD: a systematic review and meta-analysis | | 2003-2017 | | Physical injury | Children (age range: 0-19 years) | | 9 | | Systematic review and meta-analysis | | This review shows that there is a link between ADHD and poisoning in children and adolescents. | | 0.64 | | | Moderate |
| Ruiz-Goikoetxea, 2018 | | Risk of unintentional injuries in children and adolescents with ADHD and the impact of ADHD medications: a systematic review and meta-analysis. | | 1988-2017 | | Injury | Children (age range 1-18 years) | | 30 | | Systematic review and meta-analysis | | The review shows an increase in unintentional injuries in children with ADHD | | 0.68 | | | Moderate |
| Seens, 2021 | | Prevalence of bone fractures among children and adolescents with attention-deficit/hyperactivity disorder: a systematic review and meta-analysis | | 2001-2019 | | Bone fractures | Children (age range 0-19 years) | | 5 | | Systematic review and meta-analysis | | The review shows that children with ADHD experience more bone fractures | | 0.64 | | | Moderate |
| Diseases | | | | | | | | | | | | | | | | | | |
| Bellato, 2023 | | Association between ADHD and vision problems. A systematic review and meta-analysis | | 2001-2021 | | Vision problems | Children and adults | | 42 | | Systematic review | | The review shows that there is a link between ADHD and colour discrimination and contrast sensitivity, atypical accommodative response and convergence, astigmatism, hyperopia and hypermetropia, and strabismus. | | 0.95 | | | Good |
| Battison, 2023 | | Associations between Chronic Pain and Attention-Deficit Hyperactivity Disorder (ADHD) in Youth: A Scoping Review. | | 1993-2021 | | Pain | Children (age range 3-18 years) | | 11 | | Scoping review | | This review shows that there is a link between ADHD and chronic pain in children. | | 0.92 | | | Good* |
| Becker, 2022 | | ADHD and Neurodegenerative Disease Risk: A Critical Examination of the Evidence | | 2007-2021 | | Neurodegenerative Disease | Adults | | 8 | | Literature review | | This review shows that there is a link between ADHD and an increased risk for later-life development of neurogenerative diseases, especially Lewy body disease (LBD). | | 0.75 | | | Good* |
| Cortese, 2005 | | Restless legs syndrome and attention-deficit/hyperactivity disorder: A review of the literature. | | 1997-2004 | | Restless leg syndrome (RLS) | Children (age range 2-18 years) | | 8 | | Literature review | | This review shows that ADHD is linked with RLS | | 0.68 | | | Moderate* |
| Cortese, 2018 | | Association between attention deficit hyperactivity disorder and asthma: a systematic review and meta-analysis and a Swedish population-based study. | | NR | | Asthma | Children and adults | | 84 | | Systematic review and meta-analysis | | This review shows a significant association between asthma and ADHD | | 0.91 | | | Good |
| Ertürk, 2020 | | Association of ADHD and Celiac Disease: What Is the Evidence? A Systematic Review of the Literature. | | 2000-2013 | | Celiac disease | Children and adults | | 8 | | Systematic review | | The review does not show a link between ADHD and celiac disease. | | 0.5 | | | Moderate |
| Garcia-Argibay, 2023 | | The association between type 2 diabetes and attention- deficit/hyperactivity disorder: A systematic review, meta-analysis, and population-based sibling study. | | 2013-2020 | | Type 2 diabetes (T2DM) | Adults | | 4 | | Systematic review and meta-analysis | | This review shows a link between ADHD and T2DM. | | 0.86 | | | Good |
| Gaur, 2022 | | The Association between ADHD and Celiac Disease in Children | | [2000-2021](https://www.ncbi.nlm.nih.gov/pmc/articles/PMC9221618/) | | Celiac disease | Children | | 23 | | Systematic review | | This review shows that ADHD is linked with celiac disease. | | 0.65 | | | Moderate |
| Instanes, 2018 | | Adult ADHD and Comorbid Somatic Disease: A Systematic Literature Review | | 1994-2016 | | Somatic diseases | Adults | | 126 | | Systematic review | | This review showed a link between ADHD and obesity, sleep disorders, asthma, migraine and celiac disease. | | 0.6 | | | Moderate |
| Kaas, 2021 | | Association between childhood asthma and attention deficit hyperactivity or autism spectrum disorders: a systematic review with meta‐analysis | | 1995-2019 | | Asthma | Children and adults | | 25 | | Systematic review and meta-analysis | | The review shows that ADHD is linked with asthma. | | 0.95 | | | Good |
| Li 2023 | | Attention-deficit/hyperactivity disorder is associated with increased risk of cardiovascular diseases: A systematic review and meta-analysis | | 2011-2022 | | Cardiovascular disease | Children and adults | | 11 | | Systematic review and meta-analysis | | This review shoes that ADHD is associated with an increased risk for cardiovascular diseases. | | 0.95 | | | Good |
| Mahjani, 2022 | | Systematic review and meta-analysis: relationships between attention-deficit/hyperactivity disorder and urinary symptoms in children | | 2006-2018 | | Lower urinary tract symptoms | Children (age range: 4-17 years) | | 5 | | Systematic review and meta-analysis | | This review shows that children with ADHD being more likely to have lower urinary tract issues | | 0.68 | | | Moderate |
| Migueis, 2023 | | Attention deficit hyperactivity disorder and restless leg syndrome across the lifespan: A systematic review and meta-analysis | | 1996-2020 | | Restless leg syndrome (RLS) | Adults | | 24 | | Systematic review and meta-analysis | | This review shows that there is a link between ADHD and RLS. | | 0.64 | | | Moderate |
| Miyazaki, 2017 | | Allergic diseases in children with attention deficit hyperactivity disorder: a systematic review and meta-analysis | | 2010-2014 | | Allergy | Children (age range: 3-17) | | 5 | | Systematic review and meta-analysis | | This systematic review showed that children with ADHD had elevated rates of asthma. | | 0.77 | | | Good |
| Salem, 2018 | | ADHD is associated with migraine: a systematic review and meta-analysis | | 2005-2016 | | Migraine | Children and adults | | 11 | | Systematic review and meta analysis | | This review shows that there is a positive association between ADHD and migraine. | | 0.68 | | | Moderate |
| Social and lifestyle | | | | | | | | | | | | | | | | | | |
| Offending, criminality, and violence | | | | | | | | | | | | | | | | | | |
| Arrondo, 2023 | | Attention-deficit/hyperactivity disorder as a risk factor for being involved in intimate partner violence and sexual violence: a systematic review and meta-analysis | | 2001-2021 | | Violence | Adolescents and adults | | 14 | | Systematic review and meta-analysis | | Individuals with ADHD are at an increased risk of being involved in cases of violence. | | 0.86 | | | Good |
| Baggio, 2018 | | Prevalence of attention deficit hyperactivity disorder in detention settings: a systematic review and meta-analysis. | | 1985-2017 | | Prison | Children and adults | | 142 | | Systematic review and meta-analysis | | The review shows that there is a higher prevalence rate of ADHD amongst people living in detention center | | 0.82 | | | Good |
| Buitelaar, 2020 | | ADHD in Childhood and/or Adulthood as a Risk Factor for Domestic Violence or Intimate Partner Violence: A Systematic Review. | | 1998-2017 | | Violence | Adults | | 7 | | Systematic review | | This review shows that there is a link between ADHD and domestic violence or intimate partner violence. | | 0.85 | | | Good |
| Knecht, 2015 | | Attention-deficit hyperactivity disorder (ADHD), substance use disorders, and criminality: A difficult problem with complex solutions | | 1989-2014 | | Criminality | Children and adults | | 148 | | Systematic review | | This reviews shows that rate of adolescent and adult inmates with ADHD far exceeds that reported in the general population and children with ADHD have high rates of criminal behaviours. | | 0.5 | | | Moderate |
| Mohr-Jensen, 2016 | | A meta-analysis and systematic review of the risks associated with childhood attention-deficit hyperactivity disorder on long-term outcome of arrests, convictions, and incarcerations. | | 1982-2015 | | Arrest and convictions | Children and adults (age range 10-20) | | 11 | | Systematic review and meta-analysis | | This review showed that there is a link between ADHD and the estimated risks of arrests, convictions, and incarcerations in individuals. | | 0.91 | | | Good |
| Pratt, 2002 | | The Relationship of Attention Deficit Hyperactivity Disorder to Crime and Delinquency: A Meta-Analysis. | | 1976-1999 | | Crime | Adults | | 20 | | Systematic review and meta-analysis | | This review shows a link between ADHD and criminal/delinquent behaviour. | | 0.64 | | | Moderate |
| Retz, 2021 | | Attention-Deficit/Hyperactivity Disorder (ADHD), antisociality and delinquent behaviour over the lifespan | | NR | | Crime | Children and adults | | NR | | Narrative review | | This review shows that there is a link between ADHD and an increased risk for legal problems, convictions and incarcerations, and earlier onset and an increased risk for re-offending. | | 0.83 | | | Good* |
| Young, 2011 | | ADHD and offenders | | NR | | Prison | Children and adults | | NR | | Narrative review | | The review shows that youth and adults prisoners are more likely to have ADHD | | 0.5 | | | Moderate* |
| Young, 2015 | | A meta-Analysis of the prevalence of attention deficit hyperactivity disorder in incarcerated populations. | | 1985-2012 | | Prison | Children and adults | | 42 | | Systematic review and meta-analysis | | The review shows that there is a link between ADHD and incarcerated populations with a disproportionate high number of individuals with ADHD | | 0.5 | | | Moderate |
| Employment and education | | | | | | | | | | | | | | | | | | |
| Arnold, 2020 | | Long-term outcomes of ADHD: academic achievement and performance. | | NR | | Academic achievement | Children and adults | | 176 | | Systematic review | | This review shows that ADHD is linked with adverse academic outcomes. | | 0.7 | | | Good |
| Christiansen 2021 | | The impact of childhood diagnosed ADHD versus controls without ADHD diagnoses on later labour market attachment-a systematic review of longitudinal studies | | NR | | Employment | Children (age range: 6-17) | | 6 | | Systematic review | | The review shows that there is a negative link between ADHD and employment. | | 0.8 | | | Good |
| Gordon, 2019 | | The Transition of Youth with ADHD into the Workforce: Review and Future Directions. Clinical child and family psychology review | | 1961-2018 | | Education, occupation | Children and adults | | 35 | | Systematic review | | This review shows that ADHD is linked with poor educational and occupational attainments. | | 0.65 | | | Moderate |
| Polderman, 2010 | | A systematic review of prospective studies on attention problems and academic achievement | | 1985-2009 | | Academic achievement | Children (age range 4-15 years) | | 16 | | Systematic review | | The review shows that children with ADHD have lower academic achievement. | | 0.8 | | | Good |
| Varrasi, 2023 | | Schooling and Occupational Outcomes in Adults with ADHD: Predictors of Success and Support Strategies for Effective Learning. | | NR | | School | Children and adults | | NR | | Narrative review | | This review shows that adults with ADHD typically carry out a history of academic failures, with worse occupational and social functioning | | 0.67 | | | Moderate* |
| Quality of life | | | | | | | | | | | | | | | | | | |
| Agarwal, 2012 | | The quality of life of adults with attention deficit hyperactivity disorder: A systematic review | | 2005-2011 | | Quality of life | Children and adults | | 36 | | Systematic review | | This review shows that individuals with ADHD experience worse quality of life. | | 0.65 | | | Moderate |
| Danckaerts, 2010 | | The quality of life of children with attention deficit/hyperactivity disorder: A systematic review. | | NR | | Quality of life | Children and adults | | 36 | | Systematic review | | This review shows that ADHD is linked with reduced quality of life in children | | 0.7 | | | Good |
| Wanni Arachchige Dona, 2023 | | The Impact of Childhood Attention-Deficit/Hyperactivity Disorder (ADHD) on Children’s Health-Related Quality of Life: A Systematic Review and Meta-Analysis | | 2010-2022 | | Health related quality of life (HRQOL) | Children (age range 3-18 years) | | 23 | | Systematic review and meta-analysis | | The review shows that ADHD is linked with reduced health quality of life | | 1 | | | Good |
| Klassen, 2005 | | Quality of life of children with attention deficit hyperactivity disorder | | 1999-2004 | | Quality of life | Children (age range 0-18) | | 5 | | Systematic review and meta-analysis | | Children with ADHD experience lower quality of life | | 0.55 | | | Moderate |
| Lee, 2016 | | Meta-analysis of quality of life in children and adolescents with ADHD: By both parent proxy-report and child self-report | | 2004-2013 | | HRQOL | Children and adults | | 9 | | Systematic review and meta-analysis | | The review shows that there is a link between ADHD and health quality of life | | 0.86 | | | Good |
| Relationships | | | | | | | | | | | | | | | | | | |
| Ewe, 2019 | | ADHD symptoms and the teacher–student relationship: a systematic literature review. | | 2010-2016 | | Impact on relationship | Children (age range: 6-17) | | 7 | | Systematic literature review | | The review indicate students with ADHD generally feel less close to their teacher and teachers experience less emotional closeness, less co-operation and more conflicts in their relations with their students with ADHD. | | 0.85 | | | Good |
| Huynh-Hohnbaum, 2022 | | Effects of adult ADHD on intimate partnerships. | | 2015-2019 | | Relationship | Adults | | 3 | | Systematic review | | This review shows that ADHD is linked with celiac disease. | | 0.6 | | | Moderate |
| Kok, 2016 | | Problematic peer functioning in girls with ADHD: A systematic literature review | | 2001-2012 | | Peer relationship | Children-girls (age range 6-18 years) | | 13 | | Systematic review | | Yes, the review shows that girls with ADHD are more likely to experience difficulties in peer functioning | | 0.45 | | | Moderate |
| Ros, 2018 | | Social Functioning in Children With or At Risk for Attention Deficit/Hyperactivity Disorder: A Meta-Analytic Review | | NR | | Social functioning | Children | | 109 | | Meta-analysis | | This review shows a moderate association between ADHD and children’s social difficulties. | | 0.77 | | | Good |
| Soldati, 2020 | | Sexual Function, Sexual Dysfunctions, and ADHD: A Systematic Literature Review | | 1998-2019 | | Sexual function | Adults | | 11 | | Systematic review | | The review indicates that adults with ADHD report less sexual satisfaction, more sexual desire, more masturbation frequency, and more sexual dysfunctions than in the general population. | | 0.6 | | | moderate |
| Risky behaviour | | | | | | | | | | | | | | | | | | |
| Jerome, 2006** | | What we know about ADHD and driving risk: a literature review, meta-analysis and critique | | NR | | Driving risk | Children and adults | | 20 | | Literature review and meta-analysis | | This review provides evidence of a relationship between ADHD and negative driving outcome | | 0.36 | | | Poor |
| Jerome, 2006** | | Attention-deficit/hyperactivity disorder (ADHD) and driving risk: a review of the literature and a methodological critique. | | NR | | Driving risk | Children and adults | | 20 | | Literature review and meta-analysis | | This review provides evidence of a relationship between ADHD and negative driving outcome | | 0.36 | | | Poor |
| Kittel-Schneider, 2021 | | Parental ADHD in pregnancy and the postpartum period - A systematic review. | | 2002-2020 | | Pregnancy | Children (age range newborn-20 years) | | 32 | | Systematic review | | The review shows a higher risk of unplanned and teenage pregnancies in ADHD girls and women, and parental ADHD could have a negative impact on early parent-child-interaction. | | 0.65 | | | Moderate |
| Deshmukh, 2019 | | Driving and Road Rage Associated with Attention Deficit Hyperactivity Disorder (ADHD): a Systematic Review | | 2013-2019 | | Driving | Adults | | NR | | Systematic Review | | The review shows a negative impact of ADHD on drivers leading to negative driving outcomes. | | 0.36 | | | Poor |
| Roberts, 2021 | | Attention-deficit/hyperactivity disorder and risk-taking: A three-level meta-analytic review of behavioral, self-report, and virtual reality metrics | | 1998-2020 | | Risk taking | Children and adults | | 116 | | Systematic review and meta-analysis | | This review shows that ADHD increases risk-taking behaviour | | 0.55 | | | Moderate |
| Vaa, 2014 | | ADHD and relative risk of accidents in road traffic: a meta-analysis. | | 1979-2008 | | Driving | Adults | | 16 | | Systematic review and meta-analysis | | This review shows that risks of accidents were associated with comorbid Oppositional Defiant Disorder (ODD) and/or Conduct Disorder (CD), rather than with ADHD. | | 0.41 | | | Poor |
| Other/miscellaneous | | | | | | | | | | | | | | | | | | |
| Werling, 2022 | | Problematic use of digital media in children and adolescents with a diagnosis of attention-deficit/hyperactivity disorder compared to controls. A meta-analysis. | | 2013-2020 | | Problematic use of the internet | Children (mean age range 9.78-17 years) | | 14 | | Systematic review and meta-analysis | | The review shows that there is link between ADHD and time spent on digital media and severe symptoms of problematic internet use. | | 0.91 | | | Good |
| Thrower, 2020 | | Prevalence of autism spectrum disorder and attention-deficit hyperactivity disorder amongst individuals with gender dysphoria: A systematic review | | 2010-2019 | | Gender dysphoria | Children and adults | | 30 | | Systematic review | | This review suggest that ADHD is more prevalent in transgender communities | | 0.41 | | | Poor |

*Quality rating for non-systematic review was done with SANRA assessment tool

**Six reviews were included in this topic, however, two reviews were the same paper published twice under different title
